# Ethanol represses the expression of methanol-inducible genes via acetyl-CoA synthesis in the yeast *Komagataella phaffii*

**DOI:** 10.1038/s41598-018-36732-2

**Published:** 2018-12-21

**Authors:** Shin Ohsawa, Susumu Nishida, Masahide Oku, Yasuyoshi Sakai, Hiroya Yurimoto

**Affiliations:** 1 Division of Applied Life Sciences, Graduate School of Agriculture, Kyoto University, Kitashirakawa-Oiwake, Sakyo-ku, Kyoto, 606-8502 Japan; 2 Research Unit for Physiological Chemistry, the Center for the Promotion of Interdisciplinary Education and Research, Kyoto University, Kyoto, Japan

## Abstract

In methylotrophic yeasts, the expression of methanol-inducible genes is repressed by ethanol even in the presence of methanol, a phenomenon called ethanol repression. The mechanism of ethanol repression in *Komagataella phaffii* (*Pichia pastoris*) was studied, and acetyl-CoA synthesis from ethanol by sequential reactions of alcohol dehydrogenase, aldehyde dehydrogenase and acetyl-CoA synthetase (ACS) was involved in ethanol repression. Molecular analysis of the ACS-encoding gene product KpAcs1 revealed that its N-terminal motif, which is conserved in methylotrophic yeasts, was required for ethanol repression. ACS activity was downregulated during methanol-induced gene expression, which partially depended on autophagy. In addition, acetyl-CoA synthesis and phosphorylation of a transcription factor KpMxr1 were found to contribute to ethanol repression in a synergistic manner.

## Introduction

Methylotrophic yeasts, such as *Komagataella phaffii* (formerly *Pichia pastoris*), *Ogataea polymorpha* (formerly *Hansenula polymorpha*), and *Candida boidinii*, can utilize methanol as sole sources of carbon and energy. In these yeasts, methanol induces methanol metabolism-related genes as well as peroxisomes, which are highly induced during growth on methanol, and degraded by autophagy (pexophagy) after transfer of cells to glucose or ethanol medium. As such, these yeasts are used as model organisms to study peroxisome assembly and pexophagy^1–4^. In addition, heterologous gene expression systems have been developed with methylotrophic yeasts by utilizing their strong methanol-inducible promoters^5–7^.

The expression of methanol-inducible genes is strictly regulated by the carbon source. The maximum level of gene expression is achieved with methanol (methanol induction), whereas a low level of expression is observed in the absence of carbon source (derepression). Several transcription factors that regulate the expression of methanol-inducible genes have been identified and characterized. KpMit1 in *K. phaffii* (OpMpp1 in *O. polymorpha*)^8,9^, KpPrm1 (CbTrm1 in *C. boidinii*)^10,11^, and the Hap complex in *C. boidinii*^12^ are responsible for methanol induction. KpMxr1 and CbTrm2, which are homologues of *Saccharomyces cerevisiae* Adr1, are the transcription factors involved in derepression^13,14^.

**Figure 1:**
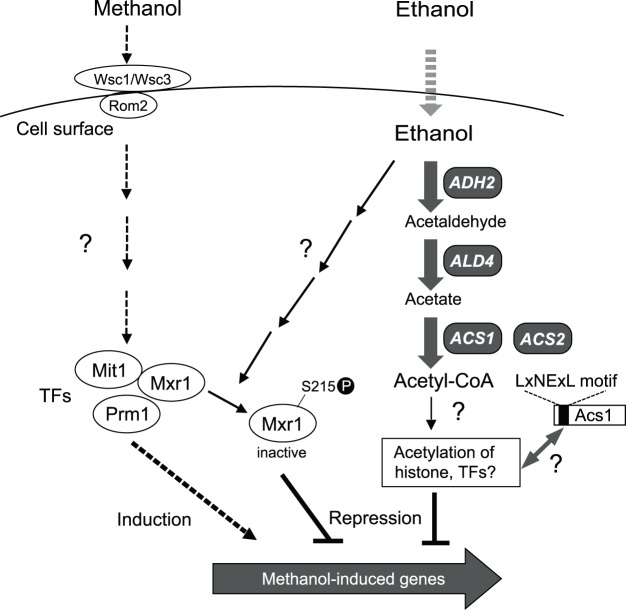
Proposed model of ethanol repression in *K. phaffii*. Cell surface sensor proteins KpWsc1 and KpWsc3, interacting with KpRom2, sense the extracellular concentration of methanol and transmit a signal to the intranuclear transcription factors (TFs), Mit1, Prm1, and Mxr1, via an uncharacterized signaling pathway, activating TFs that regulate the expression of methanol-inducible genes (left, broken arrows)^8,10,21,22^. The expression of methanol-inducible genes is repressed when methanol and ethanol are both present (ethanol repression). Acetyl-CoA, a downstream metabolite of ethanol, synthesized by the gene products of *KpADH2*, *KpALD4*, *KpACS1* and *KpACS2* (right, bold gray arrows) regulates ethanol repression in a manner distinct from the Wsc proteins-mediated expression of methanol-inducible genes. Acetyl-CoA may be utilized for acetylation of histones or other TFs that repress the expression of methanol-inducible genes (middle, solid thin arrows). KpAcs1 may supply acetyl-CoA to these proteins through interaction with the N-terminal region. In addition, Mxr1 is inactivated by phosphorylation at S215 through an unknown ethanol-sensing pathway^22^. Thus, acetyl-CoA synthesis and Mxr1 regulate ethanol repression in a synergistic manner.

The expression of methanol-inducible genes is completely repressed by glucose (glucose repression) or ethanol (ethanol repression)^15,16^. The transcription factor Mig1 is involved in glucose repression in *O. polymorpha* and *C. boidinii*^17,18^. Ethanol represses methanol-inducible gene expression even in glucose repression-insensitive mutants of *C. boidinii*^19^ and *Ogataea methanolica* (formerly *Pichia pinus*)^20^, suggesting that glucose repression and ethanol repression are mediated by distinct molecular machineries. In our previous study with *K. phaffii*^21^, we showed that the cell surface sensor proteins KpWsc1 and KpWsc3 transmit an activation signal for methanol-inducible genes through KpRom2 according to the methanol concentration (Fig. 1). We assumed that KpWsc molecules discriminate and transmit distinct signals from methanol or ethanol. KpMxr1, a positive transcription factor for methanol-inducible gene expression, is also responsible for ethanol repression through its phosphorylation^22^. In a previous study with *O. methanolica*, enzyme activity of the methanol-induced enzyme alcohol oxidase (AOX) was repressed not only by ethanol but also by acetaldehyde or acetate. These findings suggested that downstream metabolites of ethanol rather than ethanol itself repressed the expression of methanol-inducible genes, and acetyl-CoA synthetase (ACS) was not involved in ethanol repression in *O. methanolica*^23,24^. In *S. cerevisiae*, ACS partially localizes in the nucleus, and an increase in the nuclear acetyl-CoA level is involved in histone acetylation and global gene transcription^25^. In mammals, ACS was shown to directly regulate histone acetylation in neurons and spatial memory^26^.

In the previous studies using *O. methanolica*, decrease of the enzyme activity of AOX induced by methanol was investigated in the ethanol-containing media^20,23,24,27^. But the level of AOX activity is supposed to be affected not only by post-translational inactivation and proteolysis including pexophagy but also by repression of gene expression. In order to elucidate the mechanism of ethanol repression, regulation at the transcript and protein synthesis levels must be evaluated. Therefore, we assessed ethanol repression of methanol-inducible genes at the transcript and protein levels and by peroxisome formation with *K. phaffii* cells in the present study. We isolated *K. phaffii* mutants that were deficient in ethanol repression, and investigated whether acetyl-CoA synthesized from ethanol was involved in ethanol repression. In *K. phaffii*, alcohol dehydrogenase (encoded by *KpADH2*), aldehyde dehydrogenase (encoded by *KpALD4*), and ACSs (encoded by *KpACS1* and *KpACS2*), were required for ethanol repression (Fig. 1). Furthermore, the ACS enzyme activity was downregulated during induction of methanol-inducible genes. These studies shed light on the conserved molecular mechanism of ethanol repression of methanol-induced genes in methylotrophic yeasts.

## Results

### Identification of inactivated genes in ethanol repression-deficient mutants of *K. phaffii*

We derived ethanol repression-deficient mutants from the *K. phaffii* STW1 expressing green fluorescent protein (GFP) tagged with peroxisome targeting signal sequence (-SKL) under the control of the methanol-inducible *AOX1* promoter^28^. With this strain, the expression of methanol-induced genes is easily observed by the fluorescence of colonies on methanol agar medium.

We transformed strain STW1 with a linearized plasmid pREMI-Z, which can randomly insert into the chromosome of the host strain by non-homologous recombination^29^. Transformants were cultivated on ethanol plus methanol (EM) medium plates, and colonies exhibiting GFP fluorescence were identified as candidates for ethanol repression-deficient mutants. Sequence analysis of the three mutant strains revealed that pREMI-Z was inserted into the genes encoding alcohol dehydrogenase (*KpADH2*; XM_002491337.1; 74% amino acid identity with ScAdh2), acetaldehyde dehydrogenase (*KpALD4*; XM_002491373.1; 69% amino acid identity with ScAld4), and acetyl-CoA synthetase (*KpACS2*; XM_002492586.1; 69% identity with ScAcs2). In the *K. phaffii* genome, we found another ACS-encoding gene (*KpACS1;* XM_002492586.1; 67% amino acid identity with ScAcs1) (Fig. 1).

Each of our candidate genes involved in ethanol repression, *KpADH2*, *KpALD4*, *KpACS1*, and *KpACS2*, was deleted yielding *Kpadh2∆*, *Kpald4∆*, *Kpacs1∆*, and *Kpacs2∆* strains, respectively. All strains could grow on glucose, methanol, ethanol, and EM medium (Fig. S1). The fluorescence of these gene-disrupted strains was observed in EM medium. *Kpadh2∆* and *Kpald4∆* cells exhibited fluorescence and developed peroxisomes in EM medium (Fig. S2a), whereas none of the tested strains were fluorescent in ethanol medium. These findings indicated that the gene expression in the mutants was not due to aberrant signaling from ethanol but depended on methanol. Results of qRT-PCR analysis revealed that transcript levels in EM medium of all the tested methanol-inducible genes (*AOX, DAS, FLD1*, and *FDH1*) in *Kpadh2∆* and *Kpald4∆* were notably higher than those in the wild-type strain (Fig. S2b). *Kpadh2∆* cells did not exhibit GFP fluorescence in medium containing methanol and acetaldehyde or methanol and acetate, while *Kpald4∆* cells exhibited fluorescence when grown with methanol and acetaldehyde but not methanol and acetate (Fig. S2c). Therefore, the presence of a metabolite downstream of the disrupted gene product restored the ethanol repression phenotype. Similar to the regulation of alcohol oxidase activity in *O. methanolica*, ethanol and its downstream intracellular metabolites repressed the expression of methanol-inducible genes in *K. phaffii*. These results indicate that it was not ethanol itself but its intracellular metabolites that were required for ethanol repression.

### *KpACSs* are involved in ethanol repression

**Figure 2:**
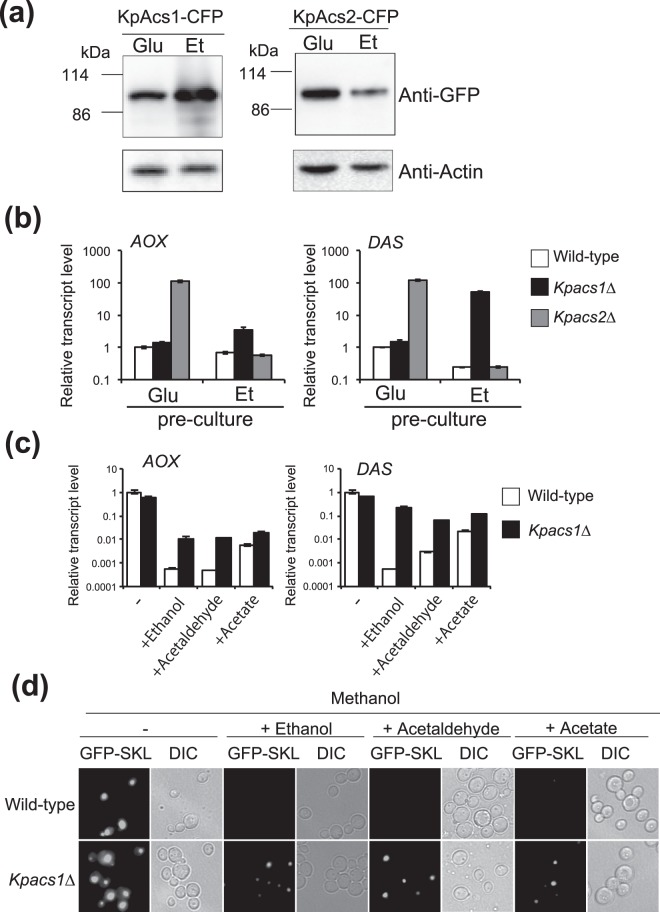
KpAcs1 and KpAcs2 are involved in ethanol repression. (**a**) Expression of KpAcs1-CFP- and KpAcs2-CFP-encoding genes under the control of each endogenous promoter. Cells cultivated in glucose (Glu) or ethanol (Et) medium of an OD_600_ of 2.0 were lysed and analyzed as described in the Materials and Methods. (**b**) Effect of the carbon source in the pre-culture on the transcript levels of methanol-inducible genes (*AOX* and *DAS*) in the wild-type, *Kpacs1∆*, and *Kpacs2∆* strains. Total mRNA was prepared from wild-type (open bars), *Kpacs1∆* (closed bars), and *Kpacs2∆* (gray bars) cells pre-cultured in glucose medium (Glu) or ethanol medium (Et), and further incubated in EM medium for 2 h. The mRNA levels were monitored by qRT-PCR analysis using the *GAP1* gene as a standard. Transcript levels are expressed as values relative to those of the wild-type strain and the means and S.D. from three independent experiments are shown. (**c**) The effect of carbon sources in addition to methanol on the transcript levels of methanol-inducible genes (*AOX* and *DAS*) in the wild-type and *Kpacs1∆* strains. Total mRNA was prepared from wild-type (open bars) and *Kpacs1∆* (closed bars) cells pre-cultured in ethanol medium and cultured in methanol medium with or without 0.5% ethanol, 0.05% acetaldehyde, or 3 mM sodium acetate (acetate) for 2 h. The mRNA levels were monitored by qRT-PCR analysis using the *GAP1* gene as the standard. Transcript levels are expressed as values relative to those of the wild-type strain, and the means and S.D. from three independent experiments are shown. (**d**) Fluorescence microscopy of wild-type and *Kpacs1∆* cells expressing GFP-SKL under the control of the *KpAOX1* promoter following pre-culture in ethanol medium, and further culture in methanol medium with or without 0.5% ethanol, 0.05% acetaldehyde, or 3 mM sodium acetate (acetate) for 4 h.

Next, we focused on the function of *KpACS1* and *KpACS2* in ethanol repression. In *S. cerevisiae, ScACS1* is induced in the presence of nonfermentable carbon sources, such as acetate or ethanol, but such regulation was not reported for *ScACS2*^30^. *KpACS1* and *KpACS2* fused to the CFP-encoding gene were expressed in *Kpacs1∆* and *Kpacs2∆* strains, respectively. The protein level of KpAcs1-CFP was found to be higher in ethanol-grown cells than in glucose-grown cells, and that of KpAcs2-CFP was higher in glucose-grown cells than in ethanol-grown cells (Fig. 2a). We determined the transcript levels of methanol-inducible genes in wild-type, *Kpacs1∆*, and *Kpacs2∆* cells that were pre-cultured in glucose or ethanol medium, and then transferred to EM medium and incubated further for 2 h. The transcript levels of methanol-inducible genes in *Kpacs2∆* cells pre-cultured in glucose medium and in *Kpacs1∆* cells pre-cultured in ethanol medium were notably higher than those in the wild-type cells (Figs 2b and S2d). These results indicate that ACS produced during pre-culturing affected ethanol repression in EM medium. When the *Kpacs2∆* strain was pre-cultured in glucose medium, the induction level of KpAcs1 was low and ethanol repression did not occur after 2-hours incubation in EM medium (Fig. 2b). Similarly, when the *Kpacs1∆* strain was pre-cultured in ethanol medium, the induction level of KpAcs2 was low and ethanol repression did not occur (Fig. 2b). These results indicate that both KpAcs1 and KpAcs2 are involved in ethanol repression. When the cells are transferred from glucose medium to EM medium, glucose remaining within cells may affect not only the regulation of methanol-inducible gene expression but also other cellular metabolisms after only 2-hours incubation in EM medium. On the other hand, glucose repression was not influenced by deletion of *KpACS1* and *KpACS2* regardless of the pre-culture conditions (Fig. S2e). Therefore, we used *Kpacs1∆* cells pre-cultured in ethanol medium in subsequent studies and focused on the function of KpAcs1 on ethanol repression.

In *Kpacs1∆* cells pre-cultured in ethanol medium, neither the expression of methanol-inducible genes nor peroxisome formation was repressed by the presence of ethanol, acetaldehyde, or acetate (Figs 2c,d and S2f). These results indicate that acetyl-CoA synthesis mediated by KpAcs1 is responsible for ethanol repression in ethanol-pre-cultured cells.

### Expression of *KpACS2*-*CFP* could not complement the function of *KpACS1* in ethanol repression

**Figure 3:**
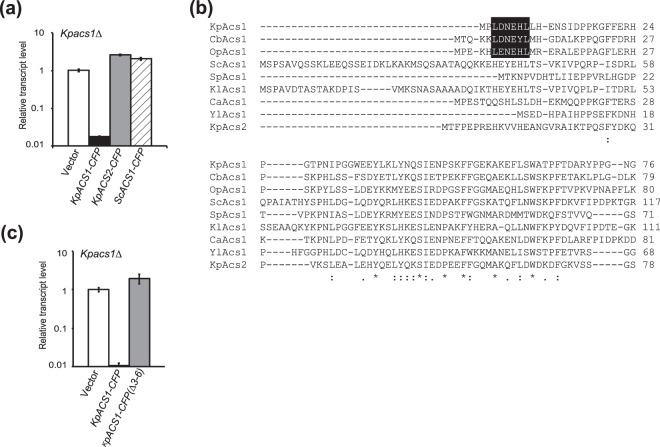
The conserved N-terminal motif of KpAcs1 is responsible for ethanol repression. (**a**) Transcript levels of *DAS* in the *Kpacs1∆* strain expressing *KpACS1-CFP, KpACS2-CFP*, or *ScACS1-CFP* under the control of the *KpACS1* promoter. Total mRNA was prepared from *Kpacs1∆* (open bars), *Kpacs1∆* cells expressing *KpACS1-CFP* (closed bars), *Kpacs1∆* cells expressing *KpACS2-CFP* (gray bars), or *Kpacs1∆* cells expressing *ScACS1-CFP* (slashed bars), following pre-culture in ethanol medium and further culture in EM medium for 2 h. The mRNA levels were monitored by qRT-PCR analysis using the *GAP1* gene as the standard. Transcript levels are expressed as values relative to those of the *Kpacs1∆* strain, and the means and S.D. from three independent experiments are shown. (**b**) Alignment of the N-terminal amino acid sequences of Asc1 homolog proteins from various yeast species. The CLUSTALW program was used to align the predicted amino acid sequences of Asc1s from *K. phaffii*, *C. boidinii*, *O. polymorpha*, *S. cerevisiae*, *Schizosaccharomyces pombe*, *Kluyveromyces lactis*, *C. albicans*, and *Yarrowia lipolytica*, and Asc2 from *K. phaffii*. The N-terminal LxNExL region (a.a. 3–8 of KpAcs1), which is conserved only in the methylotrophic yeasts, is shown with a black background. (**c**) Transcript levels of *DAS* in *Kpacs1∆* strains expressing *KpACS1-CFP* or *KpACS1(∆3–6)-CFP*. Total mRNA was prepared from *Kpacs1∆* cells (open bars), *Kpacs1∆* cells expressing *KpACS1-CFP* (closed bars), or *Kpacs1∆* cells expressing *KpACS1(∆3–6)-CFP* (gray bars) following pre-culture in ethanol medium and further culture in EM medium for 2 h. The mRNA levels were monitored by qRT-PCR analysis using the *GAP1* gene as the standard. Transcript levels are expressed as values relative to those of the *Kpacs1∆* strain, and the means and S.D. from three independent experiments are shown.

Although the *Kpacs1∆* cells retained *KpACS2*, they were impaired in ethanol repression. We assumed that pre-culturing in ethanol medium did not yield sufficient expression of *KpACS2*. Next, we expressed the *KpACS2-CFP*-encoding gene under the control of the *KpACS1* promoter in the *Kpacs1∆* strain to examine the function of KpAsc2-CFP in ethanol repression. Although *KpACS1* fused with the CFP-encoding gene (*KpACS1-CFP*) complemented the ethanol repression-deficiency of the *Kpacs1∆* strain, *KpACS2-CFP* under the control of the *KpACS1* promoter did not (Fig. 3a). Protein production of KpAcs2-CFP was confirmed by Western blot analysis and fluorescence microscopy (Fig. S3a.b). These results indicated that expression of *KpACS2* could not substitute for the function of *KpACS1* in ethanol repression under ethanol-pre-cultured conditions.

### The N-terminal conserved motif of KpAcs1 has a specific function in ethanol repression

As shown in Fig. 3a, *S. cerevisiae ACS1-CFP* under the control of the *KpACS1* promoter did not complement the ethanol repression-deficiency of the *Kpacs1∆* strain. Protein production of ScAcs1-CFP was confirmed by Western blot analysis and fluorescence microscopy (Fig. S3a,b).

The N-terminal amino acid sequence of KpAcs1 was aligned with homologs from other yeast species. We found that KpAcs1 has a LxNExL motif (a.a. 3–8 of KpAcs1) at the N-terminal region that is conserved in the methylotrophic yeasts *C. boidinii* and *O. polymorpha* (Fig. 3b). Deletion of a.a. 3–6 of KpAcs1 (KpAcs1 ∆3–6) abolished ethanol-repression in the *Kpacs1∆* strain (Fig. 3c). The protein levels of KpAcs1(∆3–6)-CFP and its fluorescence were comparable to that of KpAcs1-CFP (Fig. S3c,d). Thus, KpAcs1 was found to be responsible for ethanol repression in *K. phaffii* via its N-terminal region, which had a specific function in the regulation of the expression of methanol-inducible genes and was conserved in the methylotrophic yeasts, but not in *S. cerevisiae*.

### Autophagy is involved in downregulation of ACS for methanol induction

**Figure 4:**
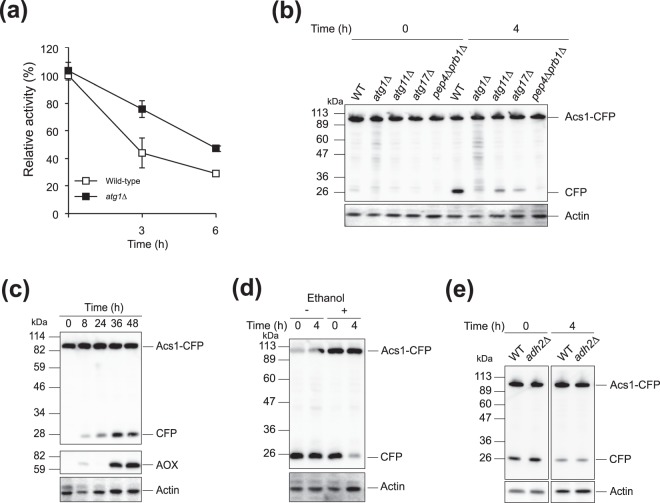
KpAcs1-CFP is the substrate of autophagy for its downregulation. (**a**) Inactivation of ACS partially depended on autophagy. The ACS activity in wild-type (open squares) and *Kpatg1∆* (closed squares) cells that were transferred from ethanol to methanol medium. The initial activity of the wild-type cells is expressed as 100%. Error bars represent S.D. of triplicate experiments. (**b**) Immunoblot detection of KpAcs1-CFP and actin in the wild-type, *Kpatg1∆, Kpatg11∆, Kpatg17∆*, and *Kppep4∆Kpprb1∆* cells at the indicated time points after transfer from ethanol medium to methanol medium. (**c**) Autophagic degradation of KpAcs1-CFP precedes induction of AOX. Immunoblot detection of KpAcs1-CFP, AOX, and actin in the wild-type cells at the indicated time points after transfer to EM medium from ethanol medium. (**d**) Addition of ethanol during methanol adaptation suppressed autophagic degradation of KpAcs1-CFP. Wild-type cells were transferred from ethanol medium to methanol medium, and cultivated for 4 h. Then, ethanol was added (+) or not (−), and cells were further incubated for 4 h. (**e**) Re-addition of ethanol terminated autophagic degradation of KpAcs1-CFP independent of KpAdh2. Wild-type and *Kpadh2∆* cells were transferred from ethanol medium to methanol medium for 4 h. Then, ethanol was added to methanol medium and the cells were harvested after 4 h.

The level of ACS activity in wild-type *K. pastoris* cells 6 h after transfer from ethanol to methanol medium decreased to ca. 40% (Fig. 4a), while in the autophagy-impaired *Kpatg1∆* strain, inactivation of ACS was delayed (Fig. 4a). Next, we investigated whether KpAcs1 was degraded by autophagy or not. If KpAsc1-CFP was delivered to the vacuole, the relatively stable CFP moiety is proteolytically detached from the fusion protein and can be detected by immunoblot analysis^31^. The wild-type and *Kpatg1∆* strains expressing the KpAcs1-CFP-encoding gene were transferred from ethanol to methanol medium. Western blot analysis revealed that CFP derived from KpAcs1-CFP was detected 4 h after transfer in the wild-type strain, but not in the *Kppep4∆prb1∆* strain, which lacks vacuolar proteases, nor in the autophagy-deficient *Kpatg1∆* strain (Fig. 4b). KpAtg11 and KpAtg17, scaffold proteins necessary for selective autophagy and bulk autophagy, respectively, were partially required for the cleavage of KpAcs1-CFP (Fig. 4b). These results indicate that KpAcs1-CFP is degraded by autophagy after transfer of cells from ethanol to methanol medium. In the *Kpatg1∆* strain, the protein level and the transcript level of AOX decreased slightly compared to those in the wild-type strain (Fig. S4a,b). Overexpression of *KpACS1-CFP* in the wild-type strain also caused a decrease in the transcript level of the methanol-induced *AOX* gene (Fig. S4b).

In EM medium, *K. phaffii* cells first utilized ethanol, and after its consumption, began to utilize methanol. Under these induction conditions, autophagic degradation of KpAcs1-CFP preceded production of AOX protein (Fig. 4c). Interestingly, autophagic degradation of KpAcs1-CFP during methanol induction in ethanol-pregrown cells was suppressed by the addition of ethanol (Fig. 4d). This response was also observed in the *Kpadh2∆* strain, indicating that the signal for autophagic degradation of KpAcs1-CFP is regulated by a mechanism distinct from that of ACS-mediated ethanol repression (Fig. 4e). These results suggest that the activity of KpAcs1 is downregulated for expression of methanol-inducible genes, in part by autophagy.

### KpMxr1 and acetyl-CoA synthesis both contribute to ethanol repression

**Figure 5:**
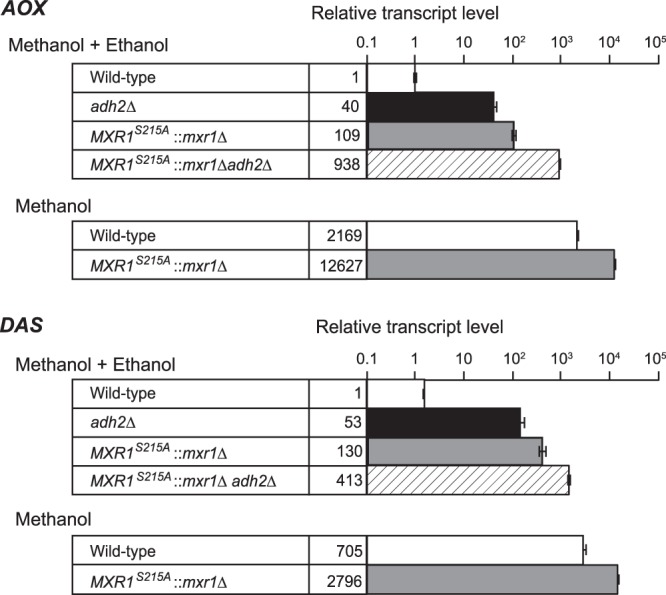
Acyl-CoA synthesis from ethanol is required for ethanol repression in coordination with phosphorylation of S215 in KpMxr1. Transcript levels of methanol-inducible genes (*AOX* and *DAS*) in the wild-type, *Kpadh2∆*, *KpMXR1*^*S215A*^::*Kpmxr1∆*, and *KpMXR1*^*S215A*^::*Kpmxr1∆Kpadh2∆* strains in EM medium or methanol medium. Total mRNA was prepared from the wild-type (open bars), *Kpadh2∆* (closed bars), *KpMXR1*^*S215A*^::*Kpmxr1∆* (gray bars), and *KpMXR1*^*S215A*^::*Kpmxr1∆Kpadh2∆* (shaded bars) strains cultured in EM medium or methanol medium for 2 h. The mRNA levels were monitored by qRT-PCR analysis using the *GAP1* gene as the standard. Transcript levels are expressed as values relative to those of the wild-type strain, and the means and S.D. from three independent experiments are shown.

KpMxr1, a transcription factor responsible for expression of methanol-inducible genes, is involved in ethanol repression in *K. phaffii*^22^. In ethanol-grown cells, KpMxr1 is inactivated by its phosphorylation at serine 215 (S215)^22^. We examined whether ethanol repression in EM medium depended on phosphorylation of S215 in KpMxr1 by expressing the KpMxr1^S215A^-encoding gene in the *Kpmxr1∆* and *Kpmxr1∆Kpadh2∆* strains. In the KpMxr1^S215A^ strain, the methanol-inducible genes *AOX* and *DAS* were highly induced in EM medium, indicating that phosphorylation of S215 in KpMxr1 is responsible for ethanol repression (Fig. 5). The induction level of methanol-inducible genes in the KpMxr1^S215A^ strain was higher than that in the *Kpadh2∆* strain in EM medium. Further deletion of *KpADH2* in the KpMxr1^S215A^ strain enhanced expression of methanol-induced genes in EM medium (Fig. 5). These results suggest that both phosphorylation of KpMxr1 and acetyl-CoA synthesis contribute to ethanol repression in a synergistic manner.

It is noteworthy that the transcript levels of methanol-induced genes in the methanol-grown KpMxr1^S215A^ strain were higher than those in the wild-type cells (Fig. 5). Therefore, phosphorylated KpMxr1 appears to regulate methanol-inducible gene expression as a negative regulator.

### Pexophagy in ethanol repression-deficient strains of *K. phaffii*

**Figure 6:**
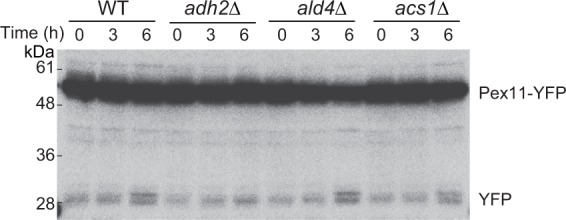
Immunoblot analysis of YFP-tagged Pex11 in wild-type, *Kpadh2∆, Kpald4∆*, and *Kpacs2∆* cells. YPD-precultured cells were transferred to methanol medium at an OD_600_ of 0.5. After 12 h, 0.5% (v/v) ethanol was added to the medium. Cells were harvested at the indicated time points after addition of ethanol.

Ethanol is known to induce the degradation of AOX via pexophagy^1^. Finally, we investigated whether deficiency in ethanol metabolism affects regulation of ethanol-induced pexophagy. The wild-type and gene disruptant strains expressing KpPex11-YFP were cultivated in methanol medium and the effect on ethanol-induced pexophagy was assessed after addition of ethanol to methanol medium. Pex11 is a peroxisomal membrane protein, and if KpPex11-YFP is delivered to the vacuole, the relatively stable YFP moiety is proteolytically detached from the fusion protein and can be detected by immunoblot analysis^32^. In the *Kpadh2∆, Kpald4∆*, and *Kpacs1∆* cells, the cleaved form of YFP gradually increased after addition of ethanol as observed in the wild-type cells (Fig. 6), indicating that ethanol-induced pexophagy is not affected by deficiency in ethanol metabolism. These results suggest that ethanol repression of methanol-inducible genes and ethanol-induced pexophagy are regulated by distinct molecular mechanisms.

## Discussion

In this study, we demonstrated that acetyl-CoA generation, catalyzed by ACS, is involved in ethanol repression of the expression of methanol-induced genes in *K. phaffii*. This yeast has two ACS-encoding genes, *KpACS1* and *KpACS2* that are mainly induced in ethanol- and glucose-grown conditions, respectively. The carbon source used for pre-culture, glucose or ethanol, was found to affect ethanol repression after transfer of cells to EM medium (Fig. 2b). This result shows that formation of acetyl-CoA from acetate is the key event for ethanol repression.

Expression of *KpACS2*, when under the control of the *KpACS1* promoter, could not replace *KpACS1* to complement the ethanol repression-deficiency of the *Kpacs1∆* strain (Fig. 3a). We found that the N-terminal region of KpAcs1, which is conserved in various genera of methylotrophic yeasts, was required for ethanol repression (Fig. 3b). We assume that KpAcs1 interacts through its N-terminal region with other factors involved in ethanol repression and has a specific role in ethanol repression other than generating acetyl-CoA. Previous studies have demonstrated the requirement of nucleocytosolic acetyl-CoA synthesized by ACS for acetylation of nucleoproteins^25^ and binding of ACS to acetyl-CoA transferase, which supplies acetyl-CoA to this enzyme^26^. Similarly, KpAcs1 may supply acetyl-CoA to other nucleocytosolic proteins, such as histones and transcription factors, through interaction with the N-terminal region (Fig. 1). Detailed studies are necessary to clarify the molecular mechanism.

In *S. cerevisiae*, the level of *ScACS1* is low in glucose medium^33^, and there have been no reports describing the regulation of *ScASC2* in ethanol medium. In the present study with *K. phaffii*, we found that ACS activity decreased after transfer of cells from ethanol to methanol medium. Inactivation of ACS activity was due in part to autophagy (Fig. 4a,b). In *K. phaffii*, autophagy is induced in cells after transfer of cells from glucose to methanol medium during lag phase (lag-phase autophagy), which partially depends on the selective autophagy factor KpAtg11 and the bulk autophagy factor KpAtg17^16^. Similarly, autophagic degradation of KpAcs1-CFP after cell transfer from ethanol to methanol medium depended on both KpAtg11 and KpAtg17 (Fig. 4b). We speculate that autophagy plays a positive role in the downregulation of KpAcs1 based on the following observations: (i) autophagic degradation of KpAcs1-CFP was observed from the very early stages of methanol induction, preceding induction of AOX (Fig. 4c); (ii) the cleaved band of KpAcs1-CFP was observed until the cells entered stationary phase (Fig. 4c). In contrast, the cleaved band of the substrate proteins of lag phase autophagy, YFP-KpAtg8 and KpAld6-CFP disappeared before the cells entered exponential phase^16^. (iii) Overexpression of *KpACS1* decreased the expression level of methanol-inducible genes, although the effect was small (Fig. S4b). We assume that autophagic degradation of ACS lowers the level of cytosolic acetyl-CoA, thereby downregulating ethanol repression.

While both the *Kpadh2∆* and KpMxr1^S215A^ strains expressed methanol-inducible genes in EM medium, the level of release from ethanol repression of the double mutant was higher than those of the single mutants (Fig. 5). Therefore, the defective effects of acetyl-CoA synthesis and phosphorylation of S215 in KpMxr1 were additive, showing that acetyl-CoA synthesis regulates ethanol repression together with KpMxr1 in a synergistic manner (Fig. 1). However, it is not known how ethanol transmits the repressing signaling to KpMxr1, and whether acetyl-CoA directly regulates KpMxr1 and other transcription factors for the expression of methanol-inducible genes or not. Further studies are needed to elucidate the molecular mechanism of ethanol repression and how acetyl-CoA regulates the expression of methanol-inducible genes.

## Materials and Methods

### Strains and media

*Escherichia coli* DH10B (Takara Bio, Otsu, Japan) was used as the host strain for plasmid DNA propagation. *E. coli* cells were grown in LB medium (1% tryptone, 0.5% yeast extract, 0.5% NaCl) at 37 °C.

The yeast strains used in this study are listed in Table S1. *K. phaffii* cells were grown on YPD (1% yeast extract, 2% peptone, 2% glucose) or YNB medium (0.67% yeast nitrogen base without amino acids). One or more of the following were used as the carbon sources in YNB medium: 2% (w/v) glucose, 1% (v/v) methanol, and 1% (v/v) ethanol. Appropriate amino acids (100 μg/ml) were added to the synthetic media. All of the components other than the carbon sources used in these media were purchased from Difco Becton Dickinson (Franklin Lakes, NJ). Growth of the yeasts was monitored by the optical density (OD) at 600 nm.

### Plasmid construction and gene disruption

The oligonucleotide primers used in this study are listed in Table S2, and plasmids are listed in Table S3. A deletion cassette for the *KpADH2* gene was constructed as follows: Primer pairs PpADH2-1-F/PpADH2-1-R and PpADH2-2-F/PpADH2-2-R were used to amplify 1.0-kb fragments using genomic DNA as the template. The primer pair PpAHD2-Zeo-F/PpADH2-Zeo-R was used to amplify a Zeocin resistance cassette using plasmid SK-Zeo^r^. Using three fragments as the template, the primer pair PpADH2-1-F/PpADH2-2-R was used to couple and amplify a 3.1-kb fragment by overlap PCR. This fragment was ligated to Topo vector pCR2.1 (Thermo Fisher Scientific, Waltham, MA), yielding the *KpADH2* disruption vector pSN100. Deletion cassettes for the *KpALD4, KpACS1, KpACS2, KpMXR1*, and *Kpatg1∆* genes were constructed similarly. Primer pairs PpALD4-1-F/PpALD4-1-R, PpALD4-2-F/PpALD4-2-R and PpALD4-Zeo-F/PpALD4-Zeo-R were used for construction of the *KpALD4* disruption vector pSN200. Primer pairs PpACS1-1-F/PpACS1-1-R, PpACS1-2-F/PpACS1-2-R, and PpACS1-Zeo-F/PpACS1-Zeo-R were for the *KpACS1* disruption vector pSN201. Primer pairs PpACS2-1-F/PpACS2-1-R, PpACS2-2-F/PpACS2-2-R, and PpACS2-Bsd-F/PpACS2-Bsd-R using plasmid pPIC6A were for the *KpACS2* disruption vector pSN101. Primer pairs PpMXR1-1-F/PpMXR1-1-R, PpMXR1-2-F/PpMXR1-2-R, and PpMXR1-Bsd-F/PpMXR1-Bsd-R using plasmid pPIC6A were for the *KpMXR1* disruption vector pSN102. Primer pairs KpnI-PpATG1-1-F/PpATG1-1-R, PpATG1-2-F/HindIII-PpATG1-2-R, and PpATG1-Bsd-F/PpATG1-Bsd-R using plasmid pPIC6A were for the *KpATG1* disruption vector pSN103.

In order to disrupt the *KpADH2, KpALD4, KpACS1, KpACS2, KpMXR1*, and *KpATG1* genes, each disruption vector, pSN100, pSN200, pSN201, pSN101, pSN102, and pSN103 respectively, was digested with SacI/XhoI, BamHI/SpeI, BamHI/XhoI, SacI/XhoI, NotI/KpnI, and KpnI/HindIII respectively, and used to transform *K. phaffii* by electroporation. Proper gene disruptions were confirmed by colony PCR.

The *KpACS1* promoter and ORF region without the STOP codon was amplified using primers KpnI-PpACS1-F/SphI-PpACS1-R. The PCR fragment was cloned into the KpnI and SphI sites of the vector pYA006, resulting in pSN400. The *KpACS1* promoter regions for coupling the *KpACS2* and *ScACS1* ORFs were amplified using primer pairs XhoI-P_*PpACS*I_-F/P_*PpACS1*_-(PpACS2)-R, and XhoI-P_*PpACS*I_-F/P_*PpACS1*_-(ScACS1)-R with genomic DNA as the template. The *KpACS2* ORF and *ScACS1* ORF regions were amplified using primer pairs (P_*PpACS1*_)-PpACS2-F/SphI-PpACS2-R and (P_*PpACS1*_)-ScACS1-F/SphI-ScACS1-R with genomic DNA as the template. Each pair of fragments was amplified and coupled by overlap PCR. Each coupled fragment was digested with KpnI and SphI and inserted into pYA006, resulting in pSN401 and pSN402. The methylotrophic yeast conserved motif in *KpACS1* was deleted using primers PpACS1-3-6d-F and PpACS1-3-6d-R for inverse PCR with pSN400 as the template, and the product was self-ligated, yielding pSN403. The *KpMXR1* promoter and ORF region without the STOP codon was amplified using primers KpnI-PpMXR1-F/BamHI-PpMXR1-R. The PCR fragment was cloned into the KpnI and BamHI sites of the vector pNT206, resulting in pSN303. pSN303 was subjected to site-directed mutagenesis by using PpMXR1-S215A-F and PpMXR1-S215A-R. The resultant plasmid was designated pSN304. The *KpACS1* ORF region without the stop codon was amplified using primers KpnI-PpACS1(ORF)-F/NotI-PpACS1-R. The PCR fragment was cloned into the KpnI and NotI sites of the vector pGAPZ A, resulting in pSN500.

### Mutagenesis and mutant screening for ethanol repression

Generation of the mutants and identification of the mutation sites were performed using the previously described gene-tagging mutagenesis method^29^. pREMI-Z was linearized with BamHI and introduced into the *K. phaffii* STW1 by electroporation (1.5 kV, 25 mF, 2 mm gap cuvettes (Bio-Rad, Hercules, CA)). Transformants were picked onto agar plates containing methanol and ethanol as carbon sources. Mutant colonies that exhibited GFP fluorescence in EM medium were selected under an LED illuminator FAS-Digi (Nippon gene, Tokyo, Japan). The genomic DNA from each mutant was digested with an appropriate restriction enzyme, self-ligated with T4 ligase, and introduced into *E. coli* DH10B cells. Clones containing recircularized plasmids were selected by growing them in low-salt LB medium (1% bactotryptone, 0.5% NaCl, 0.5% yeast extract, pH 7.5) supplemented with 25 µg/mL zeocin. For each recircularized plasmid, the *K. phaffii* genomic DNA that flanked the pREMI-Z vector was sequenced in both directions by using FW-SEQ and RV-SEQ primers. The resulting sequences were subjected to BLAST analysis using the National Center for Biotechnology Information web site (https://blast.ncbi.nlm.nih.gov/Blast.cgi).

### Fluorescence observation

*K. phaffii* cells were grown in 5 ml YPD medium to the stationary phase at 28 °C. Subsequently, 30 μl of this culture was transferred into 5 ml of fresh YPD medium and the cells were grown at 28 °C for 5 h. The culture was then harvested by centrifugation at 1,500 rpm for 5 min and the cells were transferred to 5 ml of medium and grown at 28 °C. The cells were harvested by centrifugation at the indicated time points and stored on ice until observation. Observations were carried out with an IX81 fluorescence microscope (Olympus, Tokyo, Japan). Fluorescent images were captured with a charged coupled device (CCD) camera (SenSys; PhotoMetrics, Tucson, AZ) using MetaMorph software (Universal Imaging, West Chester, PA).

### RNA isolation and quantitative reverse transcription (RT)-PCR

A single colony was inoculated into YPD medium and cultivated overnight. The cells were transferred to glucose or ethanol medium to an initial OD_600_ of 0.1 and cultivated to early exponential phase. The cells were transferred to methanol plus ethanol medium (EM medium) or methanol medium at an OD_600_ of 1.0. After 2 h, the cells were harvested by centrifugation at 10,000 × g for 1 min at 4 °C. The methods used for RNA extraction and reverse transcription were as described previously^21^.

The quantitative real time PCR (qRT-PCR) was performed with a Light Cycler Instrument (Roche Diagnostics, Basel, Switzerland). Reaction were performed with SYBR Premix Ex Taq (Takara) using the primers for *GAP1*, *AOX1*, *DAS1*, *FLD1*, and *FDH1* (Table S3). The program was as follows: 10 sec at 95 °C, 40 cycles of 5 sec at 95 °C of 20 °C/sec, 20 sec at 60 °C of 20 °C/sec. Amplicon specificity was verified by melting curve analyses conducted at 65 to 95 °C (0 sec at 95 °C of 20 °C/sec, 15 sec at 65 °C of 20 °C/sec, 0 sec at 95 °C of 0.1 °C/sec). The copy number of each sample was determined with Light Cycler software Version 4.1.

### Western blot analysis

The samples for immunoblot analyses were prepared from cells harvested at an OD_600_ of 2.0. The cells were re-suspended in 1 ml of solution I (0.2 N NaOH and 0.5% (v/v) 2-mercaptoethanol), and incubated on ice for 10 min, and then 0.1 mL of TCA solution (100% (w/v)) was added to the suspension. The lysates were centrifuged at 20,000 × *g* at 4 °C for 5 min. The supernatant was removed and the pellet was re-suspended in 1 mL of acetone by brief sonication. The obtained sample was centrifuged at 20,000 × *g* at 4 °C for 5 min, and the pellet was dried, dissolved in 80 μL of sample buffer (0.1 M Tris-HCl (pH 7.5), 2% (w/v) SDS, 1% (v/v) glycerol, 0.5% (v/v) 2-mercaptoethanol, and 0.01% (w/v) bromophenol blue) by brief sonication, and incubated at 65 °C for 10 min, then centrifuged at 20,000 × *g* for 1 min. Five μL of the supernatant was electrophoresed on a 10% SDS-PAGE gel. The proteins were transferred to a PVDF membrane by semidry blotting (ATTO, Tokyo, Japan). The blots were incubated overnight with anti-GFP antibody (JL-8; Clontech, Mountain View, CA), anti-AOX, or anti-beta actin (Abcam, Cambridge, UK) at 1:1000 dilution in TBS-T buffer. The membranes were washed 3 times with TBS-T buffer and incubated with anti-mouse-HRP (Merck Millipore, Darmstadt, Germany) or anti-rabbit-HRP at a 1:10,000 dilution for 1 h. Finally, bound secondary antibodies were detected using Western Lightning (Perkin-Elmer Life Science, Waltham, MA) and the signals were analyzed with a Light Capture system (ATTO, Tokyo, Japan). The original image data are shown in Figs S5–S12.

### Acetyl-CoA synthetase assay

The acetyl-CoA synthetase assay method was essentially as described by Jones *et al*.^34^. Samples were prepared from harvested cells that were suspended in lysis buffer [0.1 M KPB pH 7.5, 1 mM phenylmethylsulfonyl fluoride, EDTA-free complete protease inhibitor cocktail (Roche Diagnostics, Basel, Switzerland)] and lysed using Multi-Beads Shocker (Yasui Kikai, Osaka, Japan). Cell extracts were subjected to centrifugation at 10,000 × *g* for 5 min at 4 °C to remove cell debris. The reaction mixture consisted of 0.1 M KPB, pH 7.5, 5 mM MgCl_2_, 10 mM ATP, 50 mM potassium fluoride, 10 mM glutathione, 0.1 mM CoA, 10 mM potassium acetate, and 20 mM neutralized hydroxylamine. Reaction mixtures with or without ATP (control) were incubated with 0.1 mg of protein extract for 20 min at 37 °C, then 2 mL of an acidified ferric chloride solution were added. Proteins that precipitated were removed by centrifugation and the OD_546_ was determined with a spectrophotometer. Experiments were conducted in triplicate.

## Electronic supplementary material

Supplementary files

## Data Availability

The datasets generated during and/or analyzed during the current study are available from the corresponding author on reasonable request.
