# Supplementary material for: Ethanol represses the expression of methanol-inducible genes via acetyl-CoA synthesis in the yeast Komagataella phaffii
Source: Sci Rep. 2018 Dec 21;8:18051. doi: 10.1038/s41598-018-36732-2 (PMC6303403; doi:10.1038/s41598-018-36732-2)

Supplementary Information

Ethanol represses the expression of methanol-inducible genes via acetyl-CoA synthesis in the yeast *Komagataella phaffii*

Shin Ohsawa[1], Susumu Nishida[1], Masahide Oku[1], Yasuyoshi Sakai[1,2], and Hiroya Yurimoto[1,]*

[1]*Division of Applied Life Sciences, Graduate School of Agriculture, Kyoto University, Kitashirakawa-Oiwake, Sakyo-ku, Kyoto 606-8502, Japan*

[2]*Research Unit for Physiological Chemistry, the Center for the Promotion of Interdisciplinary Education and Research, Kyoto University, Japan*

*Correspondence: Hiroya Yurimoto, Division of Applied Life Sciences, Graduate School of Agriculture, Kyoto University, Kitashirakawa-Oiwake, Sakyo-ku, Kyoto 606-8502, Japan. Tel.: +81 75 753 6387; fax: +81 75 753 6454; e-mail: yury@kais.kyoto-u.ac.jp

Table S1. Yeast strains used in this study

Strain	Genotype	Reference
STW1	*arg4 his4::*P_{AOX1}*GFP-PTS1*	1
SN1100	STW1, *arg4::ARG4*	This study
SN1001	STW1, *Kpadh2Δ::Zeo*^{r}	This study
SN1101	SN1001, *arg4::ARG4*	This study
SN1002	STW1, *Kpald4Δ::Zeo*^{r}	This study
SN1102	SN1002, *arg4::ARG4*	This study
PPY12	*arg4 his4*	2
SN2100	PPY12, *arg4::ARG4*	This study
SN2101	SN2100, *his4::HIS4*	This study
SN2004	PPY12, *Kpadh2Δ::Zeo*^{r}	This study
SN2005	SN2004, *his4::HIS4*	This study
SN2006	SN2005, *arg4::ARG4*	This study
SN2003	PPY12, *Kpmxr1Δ::Bsd*^{r}	This study
SN2803	SN2003, *arg4::*P_{KpMXR1}*-MXR1*^{S215A}*-5xFLAG*	This study
SN2804	SN2803, *his4::HIS4*	This study
SN2805	SN2804, *Kpadh2Δ::Zeo*^{r}	This study
SN1003	STW1, *Kpacs1Δ::Zeo*^{r}	This study
SN1103	SN1003, *arg4::ARG4*	This study
SN1104	SN1100, *Kpacs2Δ::Bsd*^{r}	This study
SN2001	PPY12, *Kpacs1Δ::Zeo*^{r}	This study
SN2500	SN2001, *his4::HIS4*	This study
SN2501	SN2001, *his4::*P_{KpACS1}*-KpACS1-CFP*	This study
SN2503	SN2001, *his4::*P_{KpACS1}*-KpACS2-CFP*	This study
SN2505	SN2001, *his4::*P_{KpACS1}*-ScACS1-CFP*	This study
SN2502	SN2501, *arg4::ARG4*	This study
SN2504	SN2503, *arg4::ARG4*	This study
SN2506	SN2505, *arg4::ARG4*	This study
SN2507	SN2500, *arg4::ARG4*	This study
SN2508	SN2001, *his4::*P_{KpACS1}*-KpACS1(Δ3-6)-CFP*	This study
SN2509	SN2508, *arg4::ARG4*	This study
OH3001	PPY12, *Kpatg1Δ::Zeo*^{r}	This study
OH3002	OH3001, *his4::*P_{KpACS1}*-KpACS1-CFP*	This study
OH3003	OH3002, *arg4::ARG4*	This study
OH3004	PPY12, *Kpatg11Δ::Zeo*^{r}	This study
OH3005	OH3004, *his4::*P_{KpACS1}*-KpACS1-CFP*	This study
OH3006	OH3005, *arg4::ARG4*	This study
OH3007	PPY12, *Kpatg17Δ::Zeo*^{r}	This study
OH3008	OH3007, *his4::*P_{KpACS1}*-PpACS1-CFP*	This study
OH3009	OH3008, *arg4::ARG4*	This study
SMD1163	*his4 Kppep4 Kpprb1*	3
OH3010	SMD1163, *his4::*P_{KpACS1}*-KpACS1-CFP*	This study
OH3011	PPY12, *his4::HIS4*	This study
OH3012	OH3011, *arg4::*$P_{PpPex11}$*-PpPEX11-YFP*	4
OH3013	OH3012, *Ppadh2Δ::Zeo*^{r}	This study
OH3014	OH3012, *Ppald4Δ::Zeo*^{r}	This study
OH3015	OH3012, *Ppacs1Δ::Zeo*^{r}	This study
GS115	*his4*	5
SN2901	GS115, *his4::*P_{KpAOX1}*-CFP-SKL*	This study
SN2902	SN2901, *Kpatg1Δ::Bsd*^{r}	This study
SN2903	SN2901, P_{KpGPD1}*-KpACS1-Myc-6xHis::Zeo*^{r}	This study

References

1 Wiemer, E. A. C., Lüers, G., Faber, K. N., Wenzel, T., Veenhuis, M., Subramani, S. Isolation and characterization of Pas2p, a peroxisomal membrane protein essential for peroxisome biogenesis in the methylotrophic yeast *Pichia pastoris*. *J Biol Chem* **271**, 18973–18980 (1996).

2 Gould, S. J., McCollum, D., Spong, A. P., Heyman, J. A., & Subramani, S. Development of the yeast *Pichia pastoris* as a model organism for a genetic and molecular analysis of peroxisome assembly. *Yeast* **8,** 613–628 (1992).

3 Tuttle, D. L. & Dunn Jr., W. A. Divergent modes of autophagy in the methylotrophic yeast *Pichia pastoris*. *J*

Cell Sci **108**, 25-35 (1995).
4 Yamashita, S., Oku, M., Sakai, Y. & Fujiki, Y. Experimental systems to study yeast pexophagy. *Methods Mol Biol* **1595,** 249-255 (2017).
5 Cregg, J. M., Barringer, K. J., Hessler, A. Y. & Blondin, B. *Pichia pastoris* as a host system for transformations. *Mol Cell Biol* **5**, 3376-3385 (1985).

Table S2. Plasmids used in this study

Designation	Discription	Reference
SK+Zeo^r	*Zeo*^{r}	1
pPIC6A	*Bsd*^{r}	Thermo Fisher Scientific
pGAPZ A	*Zeo*^{r}, P_{GAP1}*-Myc-6xHis*	Thermo Fisher Scientific
pIB1	*HIS4*	2
pNT204	pIB1*ARG4*	3
pNT206	pIB1 *5xFLAG ARG4*	4
pSY004	pIB1 *CFP HIS4*	5
pREMI-Z	*Zeo*^{r}	6
pSN100	*ΔKpadh2::Zeo*^{r}	This study
pSN200	*ΔKpald4::Zeo*^{r}	This study
pSN201	*ΔKpacs1::Zeo*^{r}	This study
pSN101	*ΔKpacs2::Bst*^{r}	This study
pSN102	*ΔKpmxr1::Bst*^{r}	This study
pSN103	*ΔKpatg1*::*Bsd*^{r}	This study
pSY8601	*ΔKPpatg1*::Zeo^r	5
pSY8611	*ΔKpatg11*::Zeo^r	5
pSY8617	*ΔKpatg17*::Zeo^r	5
pYA006	P_{AOX1}*CFP-SKL HIS4*	7
pSN400	P_{KpACS1}*KpACS1-CFP HIS4*	This study
pSN401	P_{KpACS1}*KpACS2-CFP HIS4*	This study
pSN402	P_{KpACS1}*ScACS2-CFP HIS4*	This study
pSN403	P_{KpACS1}*KpACS1(Δ3-6)-CFP HIS4*	This study
pSN303	P_{KpMXR1}*KpMXR1-5xFLAG ARG4*	This study
pSN304	P_{KpMXR1}*KpMXR1*^{S215A}*-5xFLAG ARG4*	This study
pSN500	*Zeo*^{r}, P_{GAP1}*-KpACS1-Myc-6xHis*	This study

References

1 Yano, T., Takigami, E., Yurimoto, H. & Sakai, Y. Yap1-regulated glutathione redox system curtails accumulation of formaldehyde and reactive oxygen species in methanol metabolism of *Pichia pastoris*. *Eukaryot Cell* **8**, 540-549 (2009).

2 Sears, I. B., O'Connor, J., Rossanese, O. W. & Glick, B.S. A versatile set of vectors for constitutive and regulated gene expression in *Pichia pastoris*. *Yeast* **14**, 783-790 (1998).

3 Tamura, N., Oku, M. & Sakai, Y. Atg8 regulates vacuolar membrane dynamics in a lipidation-independent manner in *Pichia pastoris*. *J Cell Sci* **123**, 4107-4116 (2010).

4 Tamura, N., *et al.* Atg18 phosphoregulation controls organellar dynamics by modulating its phosphoinositide-binding activity. *J Cell Biol* **202**, 685-698 (2013).

5 Yamashita, S. *et al.* Lag-phase autophagy in the methylotrophic yeast *Pichia pastoris*. *Genes Cells* **14**, 861-870 (2009).

6 Mukaiyama, H. *et al.* Paz2 and 13 other *PAZ* gene products regulate vacuolar engulfment of peroxisomes during micropexophagy. *Genes Cells* **7**, 75-90 (2002).

7 Ano, Y. *et al.* A sorting nexin PpAtg24 regulates vacuolar membrane dynamics during pexophagy via binding to phosphatidylinositol-3-phosphate. *Mol Biol Cell* **16**, 446-457 (2005).

Table S3. Primers used in this study

Designation	DNA Sequence
PpADH2-1-F	5'-CCCGAGCTCCAAATTGCACGACCAGAGTG-3'
PpADH2-1-R	5'-TTTGAAGCTATGGTGTGTGGGCAATAGGCAGGGTGAAAGGA-3'
PpADH2-2-F	5'-CGAAGGCTTTAATTTGCAAGCTCTGTGATCAAACCGTTGTGG-3'
PpADH2-2-R	5'-CCGCTCGAGATGGTACCCAGGCAAAGAGA-3'
PpAHD2-Zeo-F	5'-TCCTTTCACCCTGCCTATTGCCCACACACCATAGCTTCAAA -3'
PpADH2-Zeo-R	5'-CCACAACGGTTTGATCACAGAGCTTGCAAATTAAAGCCTTCG -3'
PpALD4-1-F	5'-CGGGATCCTGTCAGTTCCAGACCAGCAG-3'
PpALD4-1-R	5'-TTTGAAGCTATGGTGTGTGGGTTGGGCAAGGAAAAATCAAG-3'
PpALD4-2-F	5'-CGAAGGCTTTAATTTGCAAGCTTGGGTCAACACCTACAACGA-3'
PpALD4-2-R	5'-GACTAGTTCCTCGTCCTCGACAAAGTT-3'
PpALD4-Zeo-F	5'-CTTGATTTTTCCTTGCCCAACCCACACACCATAGCTTCAAA -3'
PpALD4-Zeo-R	5'-TCGTTGTAGGTGTTGACCCAAGCTTGCAAATTAAAGCCTTCG -3'
PpACS1-1-F	5'-AACTGCAGGCTCAGCGGTTATGTTGGTT-3'
PpACS1-1-R	5'-TTTGAAGCTATGGTGTGTGGGACGAGCGTCAGTGAAAGGAG-3'
PpACS1-2-F	5'-CGAAGGCTTTAATTTGCAAGCTTGTCAGAAAAGAGATTGGACCAT-3'
PpACS1-2-R	5'-GACTAGTCAAAACCTTTTGATCATACTGTGG-3'
PpACS1-Zeo-F	5'-CTCCTTTCACTGACGCTCGTCCCACACACCATAGCTTCAAA -3'
PpACS1-Zeo-R	5'-ATGGTCCAATCTCTTTTCTGACAAGCTTGCAAATTAAAGCCTTCG -3'
PpACS2-1-F	5'-TGGTTGGCATTTATTCACTGTC-3'
PpACS2-1-R	5'-GAAGCTATGGTGTGTGGGCTATGACCAGTGCCTCGAAAAT-3'
PpACS2-2-F	5'-CGAAGGCTTTAATTTGCAAGCTCCGTCAGCGCCTATCTAATC-3'
PpACS2-2-R	5'-TTAATCGTACGGTGCAACCA-3'
PpACS2-Bsd-F	5'-ATTTTCGAGGCACTGGTCATAGCCCACACACCATAGCTTC -3'
PpACS2-Bsd-R	5'-GATTAGATAGGCGCTGACGGAGCTTGCAAATTAAAGCCTTCG-3'
PpMXR1-1-F	5'-TCAAAAAGCCAAAGGACCAG-3'
PpMXR1-1-R	5'-GAAGCTATGGTGTGTGGGCTTGCGTGGGATAAAGTCATCA-3'
PpMXR1-2-F	5'-CGAAGGCTTTAATTTGCAAGCTTTCTGGATTCTTCTTCCTTTGT -3'
PpMXR1-2-R	5'-AGTTTCCTTCCAGCGGAGTT-3'
PpMXR1-Bsd-F	5'-TGATGACTTTATCCCACGCAAGCCCACACACCATAGCTTC-3'
PpMXR1-Bsd-R	5'-ACAAAGGAAGAAGAATCCAGAAAGCTTGCAAATTAAAGCCTTCG-3'
KpnI-PpACS1-F	5'-GGGGTACCCAGCAAAATCATCTGGCTCA-3'
SphI-PpACS1-R	5'-ACATGCATGCTTTGCGGGCATCCCTTTTAA-3'
XhoI-P_{PpACS1}-F	5'-CTCGAGCAGCAAAATCATCTGGCTCA-3'
P_{PpACS1}-(PpACS2)-R	5'-CTCTTGGCTCTGGAAAAGTCATAATTGATCAACAACTAAGTCGTATCCT-3'
P_{PpACS1}-(ScACS1)-R	5'-GTACGGCAGAGGGCGACATAATTGATCAACAACTAAGTCGTATCCT-3'
(P_{PpACS1})-PpACS2-F	5'-AGGATACGACTTAGTTGTTGATCAATTATGACTTTTCCAGAGCCAAGAG-3'
SphI-PpACS2-R	5'-GCATGCCTTCTTGAAGAACTGGTTATCAACAG-3'
(P_{PpACS1})-ScACS1-F	5'-AGGATACGACTTAGTTGTTGATCAATTATGTCGCCCTCTGCCGTAC-3'
SphI-ScACS1-R	5'-GCATGCCAACTTGACCGAATCAATTAGATG-3'
PpACS1-3-6d-F	5'-TATGCCACACTTACTTCATGAAAAT-3'
PpACS1-3-6d-R	5'-AGTAAGTGTGGCATAATTGATCAACA-3'
KpnI-PpMXR1-F	5'-GGGGTACCGGTGTCATCAAGTGGCGTACTG -3'
BamHI-PpMXR1-R	5'-CGGGATCCGACACCACCATCTAGTCGGTTT-3'
PpMXR1-S215A-F	5'-AGAGCTGCTTTCTCCGCCGTTAGTGGA-3'
PpMXR1-S215A-R	5'-GGAGAAAGCAGCTCTTCTTAGTCCAAG-3'
KpnI-PpACS1(ORF)-F	5'-GGGGTACCATGCCATTAGATAACGAACACTTACTT-3'
NotI-PpACS1-R	5'-GCGGCCGCGTTTGCGGGCATCCCTTTTAA-3'
KpnI-PpATG1-1-F	5'-GGGGTACCCAATTGCACCGTTAACATCG-3'
PpATG1-1-R	5'-GAAGCTATGGTGTGTGGGCTTGCTCACAATCCAATAGAGCAA -3'
PpATG1-2-F	5'-CGAAGGCTTTAATTTGCAAGCTGGCTATCTGGATGCTGGAAG -3'
HindIII-PpATG1-2-R	5'-CCCAAGCTTTAGGAAGGGGTTCCTTGGTT-3'
PpATG1-Bsd-F	5'-TTGCTCTATTGGATTGTGAGCAAGCCCACACACCATAGCTTC-3'
PpATG1-Bsd-R	5'-CTTCCAGCATCCAGATAGCCAGCTTGCAAATTAAAGCCTTCG -3'
FW-SEQ	5'-GTGGAACGAAAACTCACGTTAAGGGAT-3'
RV-SEQ	5'-GAGTAAAAAAGGAGTACAAACATTTTGAAGCTATGGTG-3'
RT-AOX1-F	5'-AGGGCTTCTGAGTCCCAAGG-3'
RT-AOX1-R	5'-AGCAGAGTCGGAACGACGAC-3'
RT-DAS1-F	5'-TTGCGTATGGCTGCTCTTCA-3'
RT-DAS1-R	5'-GGGTTGGACCATCTTCACCA-3'
RT-FLD1-F	5'-CACGCTTTCTGGTGCAGATG-3'
RT-FLD1-R	5'-ATCCCCAACCTTCACGGACT-3'
RT-FDH1-F	5'-GCACATTCCTGACGCTGATG-3'
RT-FDH1-R	5'-GACACCAGCAACGACCAACA-3'
RT-ACS1-F	5'-CTGACGCTCGTTACCCACCT-3'
RT-ACS1-R	5'-CGCGTTCAACTCACCATTCA-3'
RT-GAP1-F	5'-CCACCGGTGTTTTCACCACT-3'
RT-GAP1-R	5'-CACCGACAACGAACATTGGA-3'

Legends to Supplementary Figures

Fig. S1. Growth of the wild-type, *Kpadh2*Δ*, Kpald4*Δ*, Kpacs1*Δ*,* and *Kpacs2*Δ strains on glucose, ethanol, methanol, and EM media. Serial dilutions of cell suspension of each strain were dropped onto YNB agar plates containing indicated carbon sources. Cell growth was scored after 3-days incubation at 28°C.

Fig. S2. Ethanol metabolism is involved in the repression of expression of methanol-induced genes. (**a**) Fluorescence microscopy of the wild-type, *Kpadh2*Δ*,* and *Kpald4*Δ cells expressing GFP-SKL under the control of the *KpAOX1* promoter in EM medium, ethanol medium, and methanol medium, (**b**) Transcript levels of methanol-inducible genes in the wild-type, *Kpadh2*Δ, and *Kpald4*Δ strains. Total mRNA was prepared from the wild-type (open bars), *Kpadh2*Δ (closed bars), and *Kpald4*Δ (gray bars) cells incubated in EM medium or methanol medium for 2 h. The mRNA levels were monitored by qRT-PCR analysis using the *GAP1* gene as the standard. Transcript levels are expressed as values relative to those of the wild-type strain, and the means and S.D. from three independent experiments are shown. (**c**) Fluorescence microscopy of the wild-type, *Kpadh2*Δ*,* and *Kpald4*Δ cells expressing GFP-SKL under the control of the *KpAOX1* promoter in methanol medium with 0.05% acetaldehyde or 3 mM sodium acetate (acetate). (**d**) Transcript levels of *KpFLD1* and *KpFDH1* in the wild-type, *Kpacs1*Δ, and *Kpacs2*Δ strains. Total mRNA was prepared from the wild-type (open bars), *Kpacs1*Δ (closed bars), and *Kpacs2*Δ (gray bars) cells pre-cultured in glucose medium (Glu) or ethanol medium (Et) and cultured in EM medium for 2 h. The mRNA levels were monitored by qRT-PCR analysis using the *GAP1* gene as the standard. Transcript levels are expressed as values relative to those of the wild-type strain, and the means and S.D. from three independent experiments are shown. (**e**) Transcript levels of methanol-inducible genes in the wild-type, *Kpacs1*Δ, and *Kpacs2*Δ strains. Total mRNA was prepared from the wild-type (open bars), *Kpacs1*Δ (closed bars), and *Kpacs2*Δ (gray bars) cells pre-cultured in glucose medium (Glu) or ethanol medium (Et) and cultured in the medium containing glucose and methanol for 2 h. The mRNA levels were monitored by

qRT-PCR analysis using the *GAP1* gene as the standard. Transcript levels are expressed as values relative to those of the wild-type strain, and the means and S.D. from three independent experiments are shown. (**f**) Transcript levels of *KpFLD1* and *KpFDH1* in the wild-type and *Kpacs1*Δ strains precultured in ethanol medium and cultured in methanol medium with or without 0.5% ethanol, 0.05% acetaldehyde, or 3 mM sodium acetate (acetate) for 2 h. Total mRNA was prepared from the wild-type (open bars) and *Kpacs1*Δ (closed bars) cells. The mRNA levels were monitored by qRT-PCR analysis using the *GAP1* gene as the standard. Transcript levels are expressed as values relative to those of the wild-type strain, and the means and S.D. from three independent experiments are shown.

Fig. S3. Expression of the Acs-CFP-encoding genes under the control of the *KpACS1* promoter in EM medium. (**a**) Fluorescence microscopy of the *Kpacs1*Δ strain expressing *KpACS1-CFP*, *KpACS2-CFP*, or *ScACS1-CFP*. Cells were transferred from ethanol medium to EM medium for 2 h. (**b**) Production of KpAcs1-CFP, KpAcs2-CFP, and ScAcs1-CFP proteins in *K. phaffii*. Cultures that reached an OD_{610} of 2.0 were lysed and analyzed by immunoblot analysis as described in Materials and Methods. (**c**) Fluorescence microscopy of the *Kpacs1*Δ strain producing KpAcs1-CFP or KpAcs1(Δ3-6)-CFP. Cells were transferred from ethanol medium to EM medium for 2 h. (**d**) Production of KpAcs1-CFP and KpAcs1(Δ3-6)-CFP proteins. Cultures that reached an OD_{610} of 2.0 were lysed and analyzed by immunoblot analysis as described in Materials and Methods.

Fig. S4. Downregulation of KpAcs1 by autophagy supports the expression of methanol-inducible genes. (**a**) Immunoblot detection of KpAcs1-CFP and AOX in wild-type and *Kpatg1*Δ cells at the indicated time points after transfer from ethanol to methanol medium. The band positions corresponding to KpAcs1-CFP and its processed form (CFP) are indicated. (**b**) Overexpression of *KpACS1* lowered the expression of methanol-inducible genes. Transcript levels of *AOX* in the wild-type strain, the wild-type strain expressing *KpACS1-Myc-6xHis* under the control of the *KpGAP1* promoter (+*KpACS1*)*,* and the *Kpatg1*Δ strain expressing *KpACS1-Myc-6xHis* under the control of the *KpACS1* promoter

(*atg1*Δ). Total mRNA was prepared from these strains after pre-culturing in ethanol medium and culturing in methanol medium for 2 h. The mRNA levels were monitored by qRT-PCR analysis using the *GAP1* gene as the standard. Transcript levels are expressed as the values (%) relative to those of the wild-type strain, and the means and S.D. from three independent experiments are shown.

Fig. S5. The original image data of Western blot analysis in the left panel of Fig. 2a. The same samples were loaded on different gels. For detection of actin, a portion of the gel was cut off. Detection of secondary antibodies was done at the same time.

Fig. S6. The original image data of Western blot analysis in the right panel of Fig. 2a. After electrophoresis, the gel was cut off into two. The upper part of the gel was used for detection of CFP and the lower part of the gel was used for detection of actin. Detection of secondary antibodies was done at the same time. For the detection of actin (lower panel), exposure time was longer than that for the detection of CFP (upper panel).

Fig. S7. The original image data of Western blot analysis in Fig. 4b. The same samples were loaded on different gels. For detection of actin (lower panel), a portion of the gel was cut off.

Fig. S8. The original image data of Western blot analysis in Fig. 4c. The same samples were loaded on different gels. For detection of actin (lower panel), a portion of the gel was cut off.

Fig. S9. The original image data of Western blot analysis in Fig. 4d and Fig. S3b. The same samples were loaded on different gels. For detection of actin, a portion of the gel was cut off. Detection of secondary antibodies was done at the same time. For the detection of actin (lower panel), exposure time was longer than that for the detection of CFP (upper panel).

Fig. S10. The original image data of Western blot analysis in Fig. 4e. The same samples were loaded on different gels. For detection of actin (lower panel), a portion of the gel was cut off.

Fig. S11. The original image data of Western blot analysis in Fig. 6.

Fig. S12. The original image data of Western blot analysis in Fig. S3d. The same samples were loaded on different gels. For detection of actin, a portion of the gel was cut off. Detection of secondary antibodies was done at the same time. For the detection of actin (lower panel), exposure time was longer than that for the detection of CFP (upper panel).

Fig. S13. The original image data of Western blot analysis in Fig. S4a. The same samples were loaded on different gels. For detection of AOX (upper panel) and actin (lower panel), a portion of the gel was cut off. For detection of CFP and AOX (upper panel), detection of secondary antibodies was done at the same time.

Supplementary Fig. S1

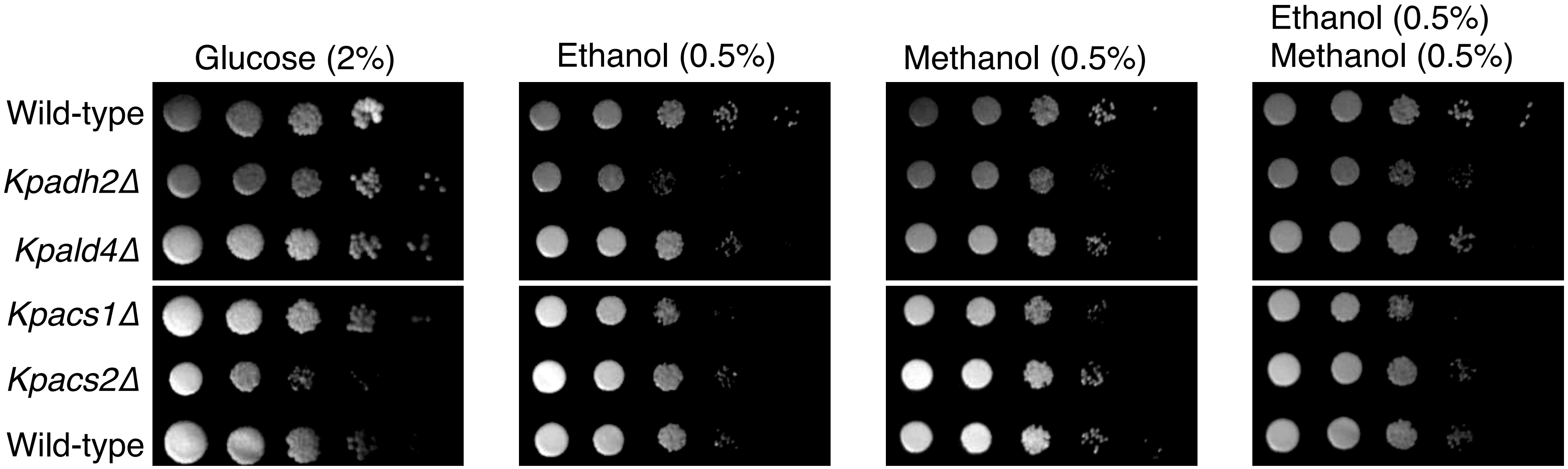

Supplementary Fig. S2

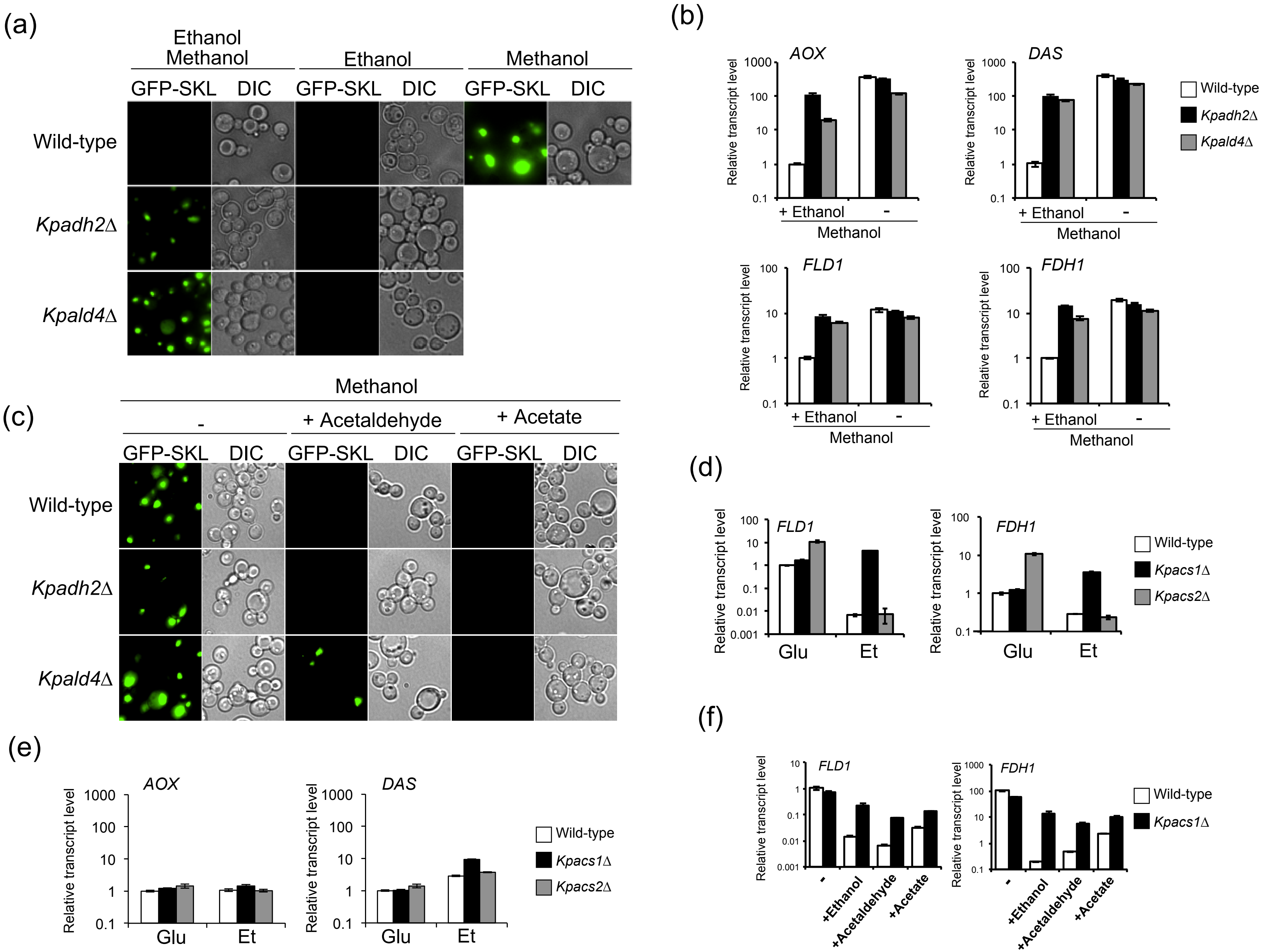

Supplementary Fig. S3

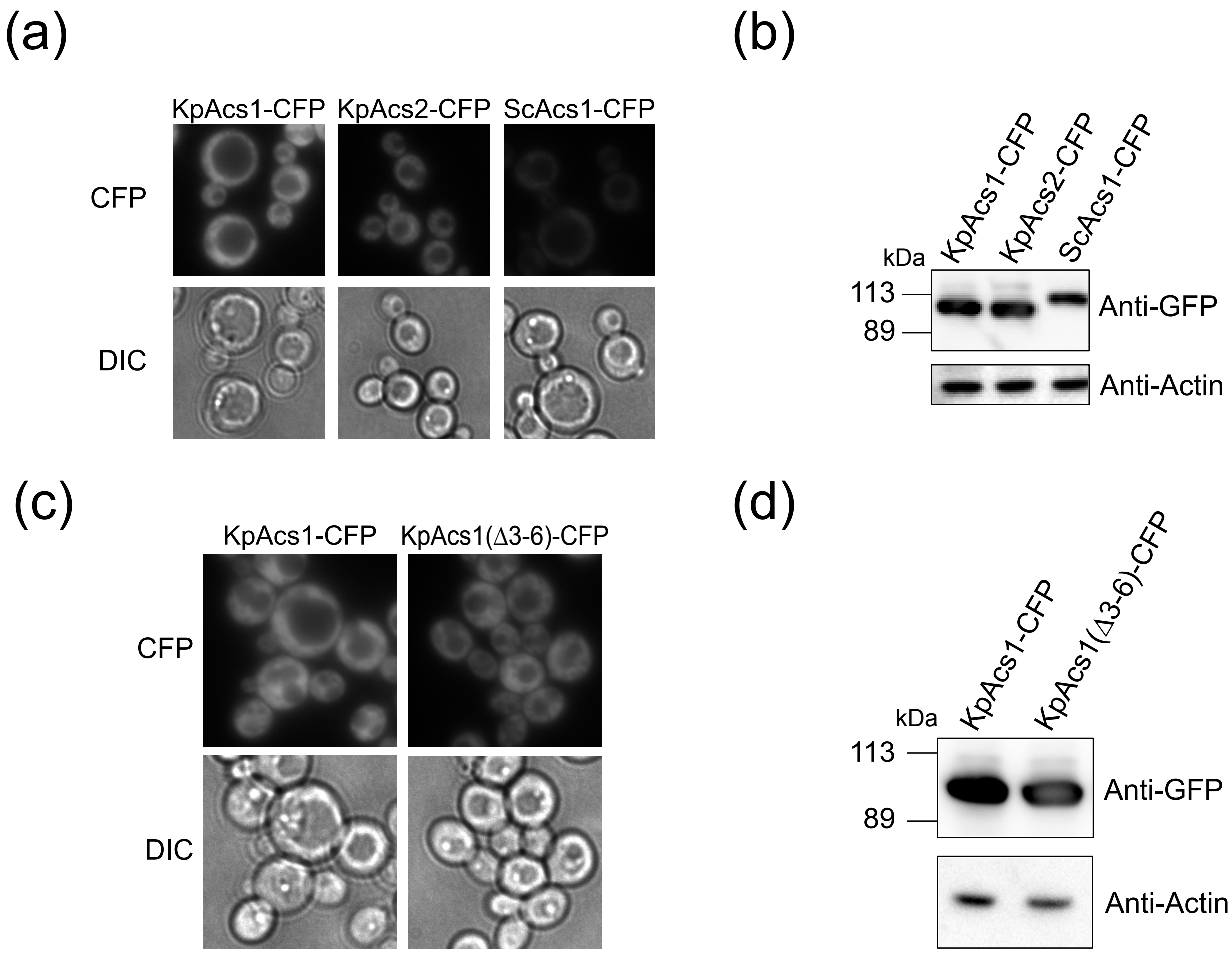

Supplementary Fig. S4

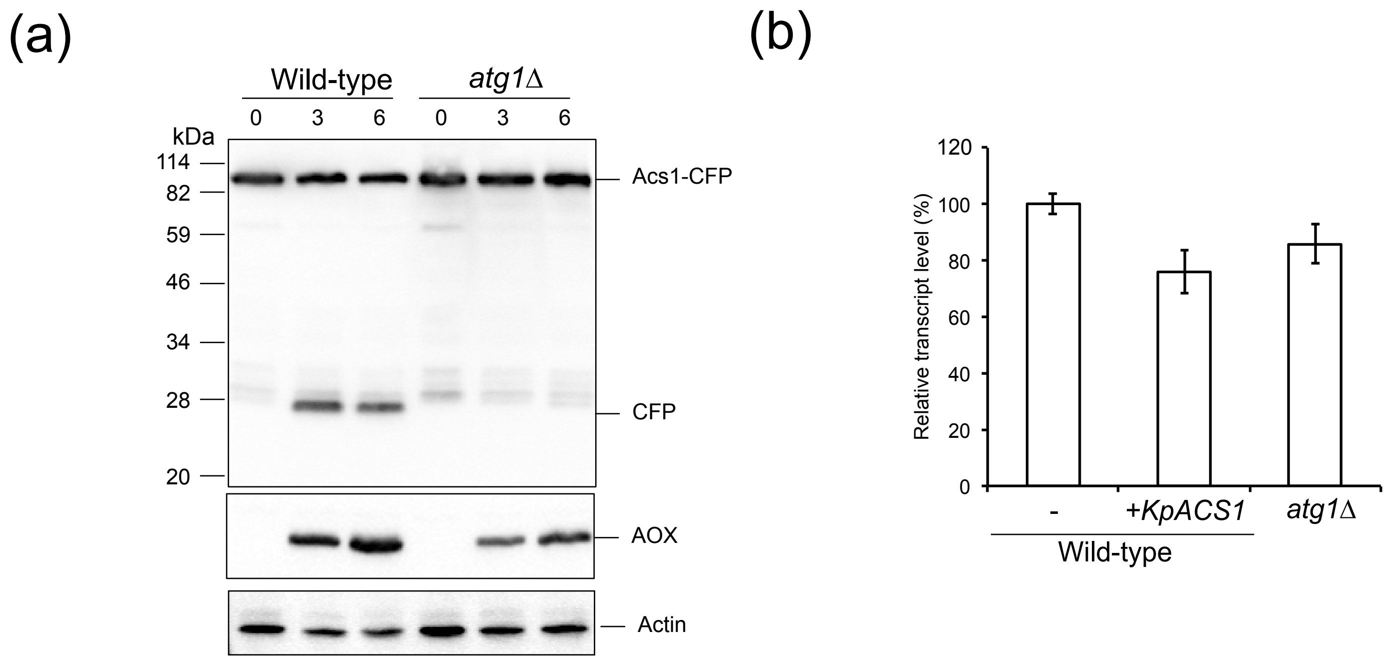

Supplementary Fig. S5

Fig. 2a, left; anti-GFP& anti-Actin

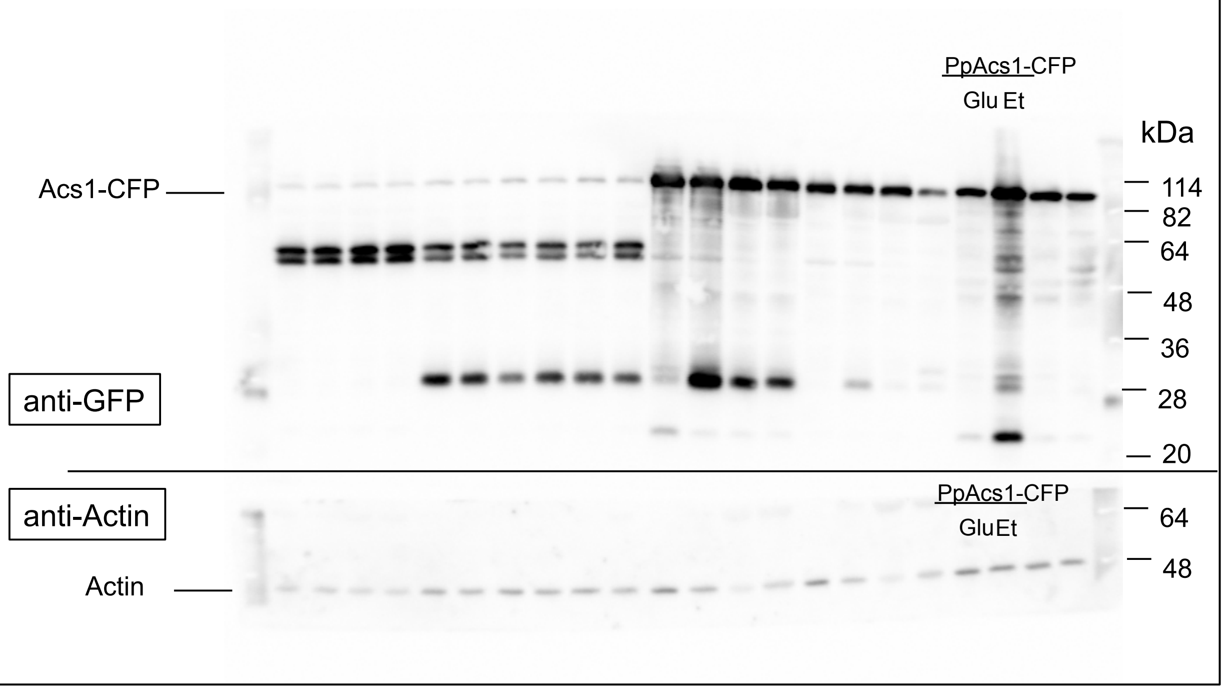

Supplementary Fig. S6

Fig. 2a, right; anti-GFP

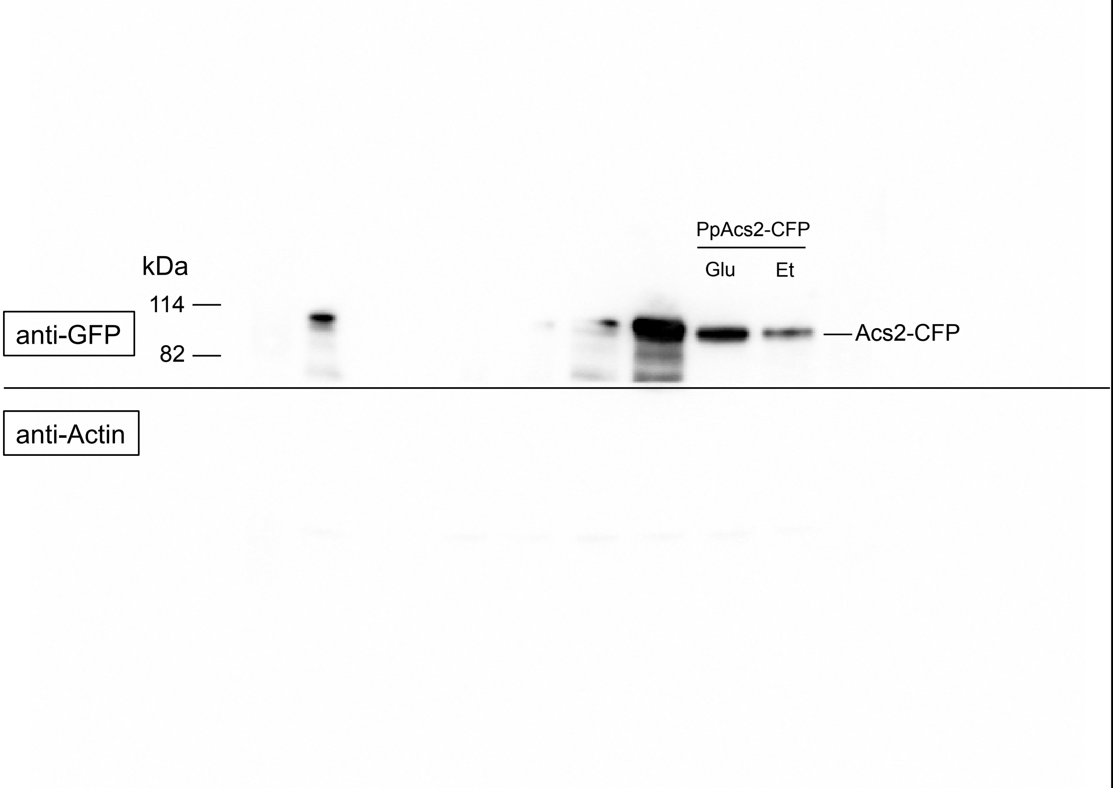

Fig. 2a, right; anti-Actin

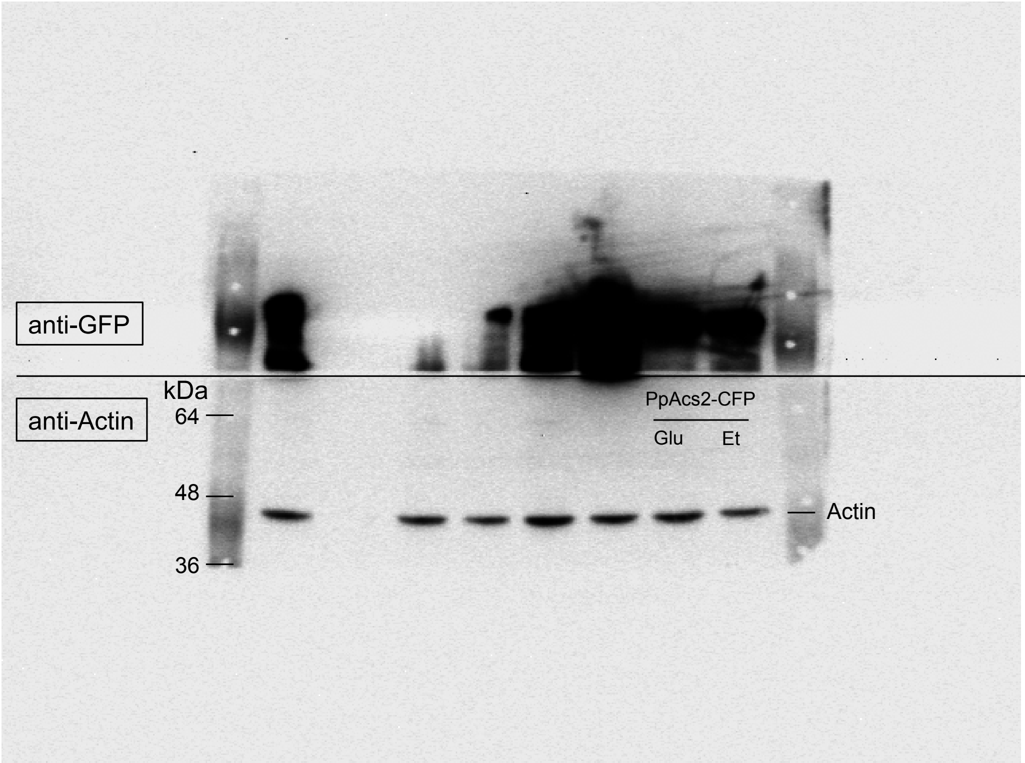

Supplementary Fig. S7

Fig. 4b; anti-GFP

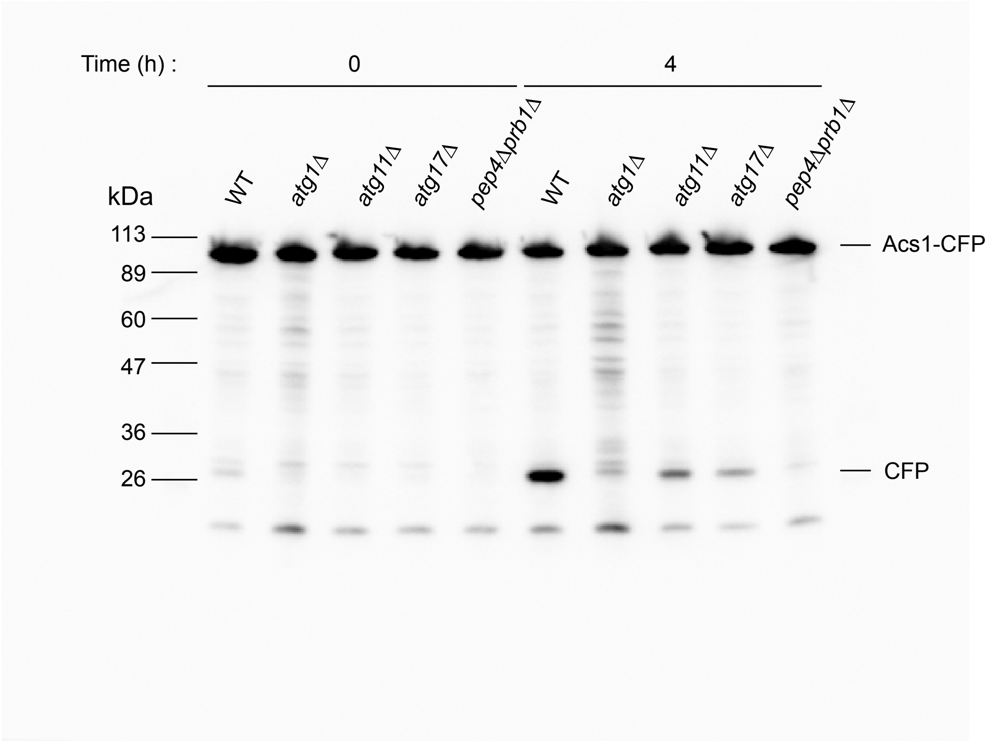

Fig. 4b; anti-Actin

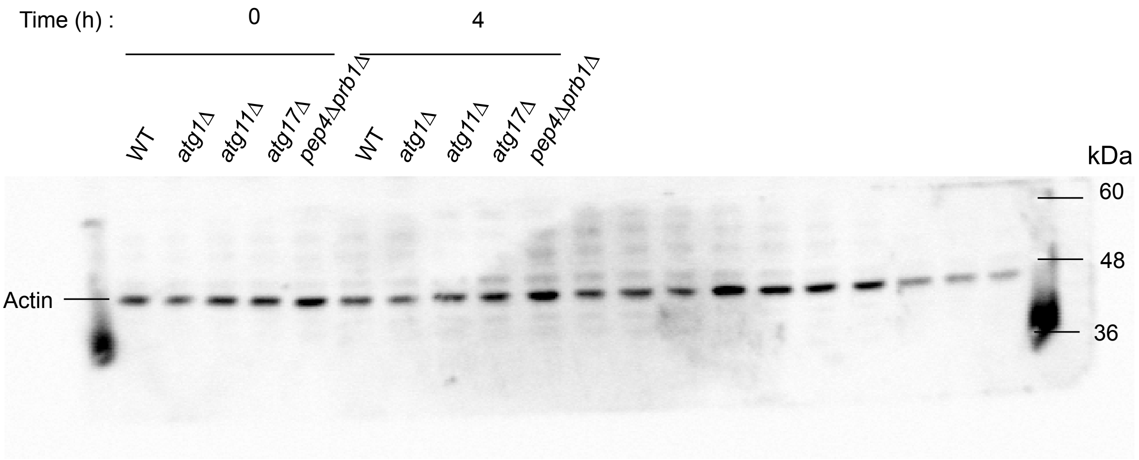

Supplementary Fig. S8

Fig 4c; anti-AOX, anti-GFP

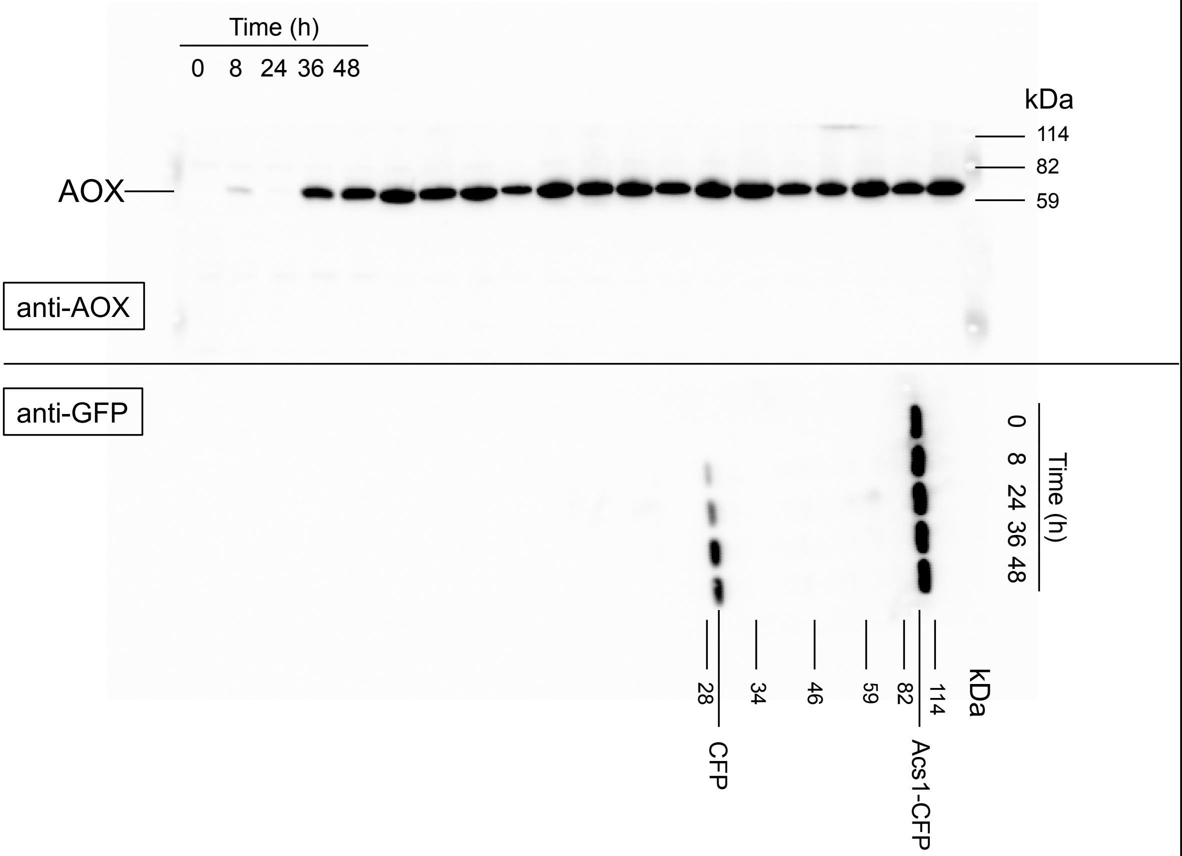

Fig. 4c; anti-Actin

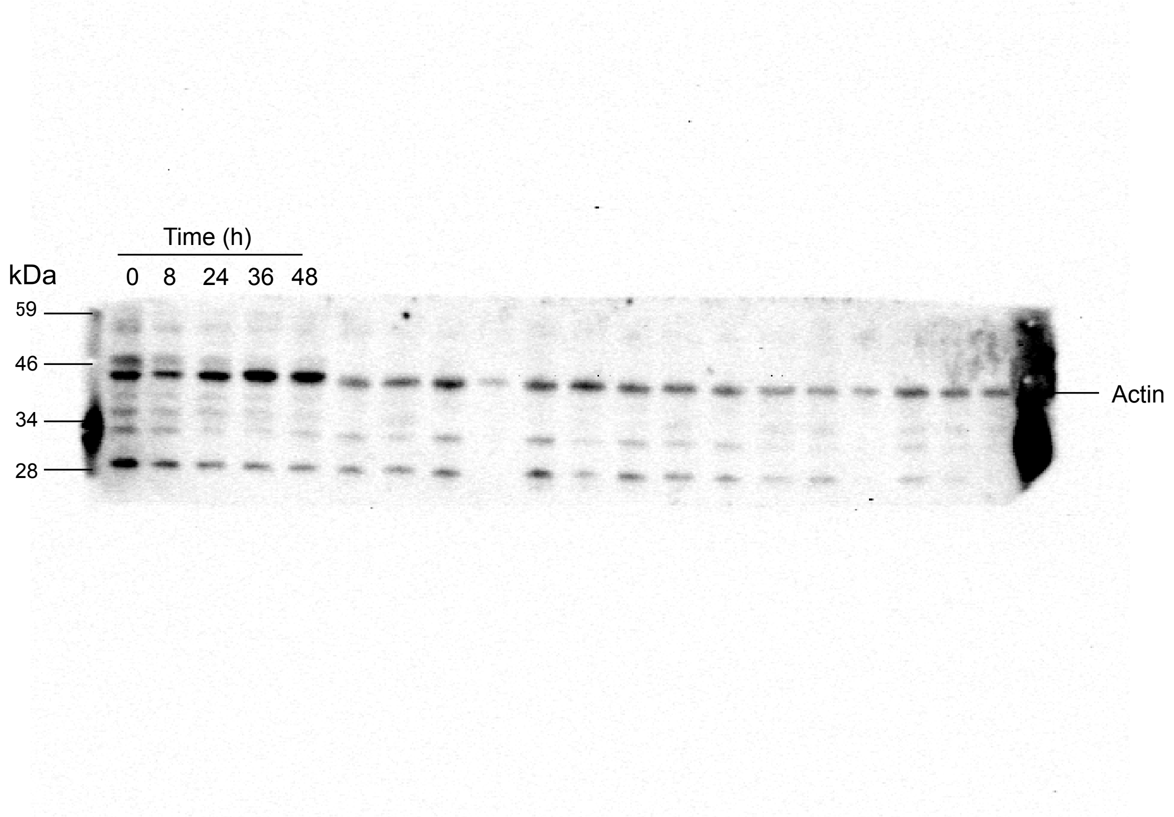

Supplementary Fig. S9

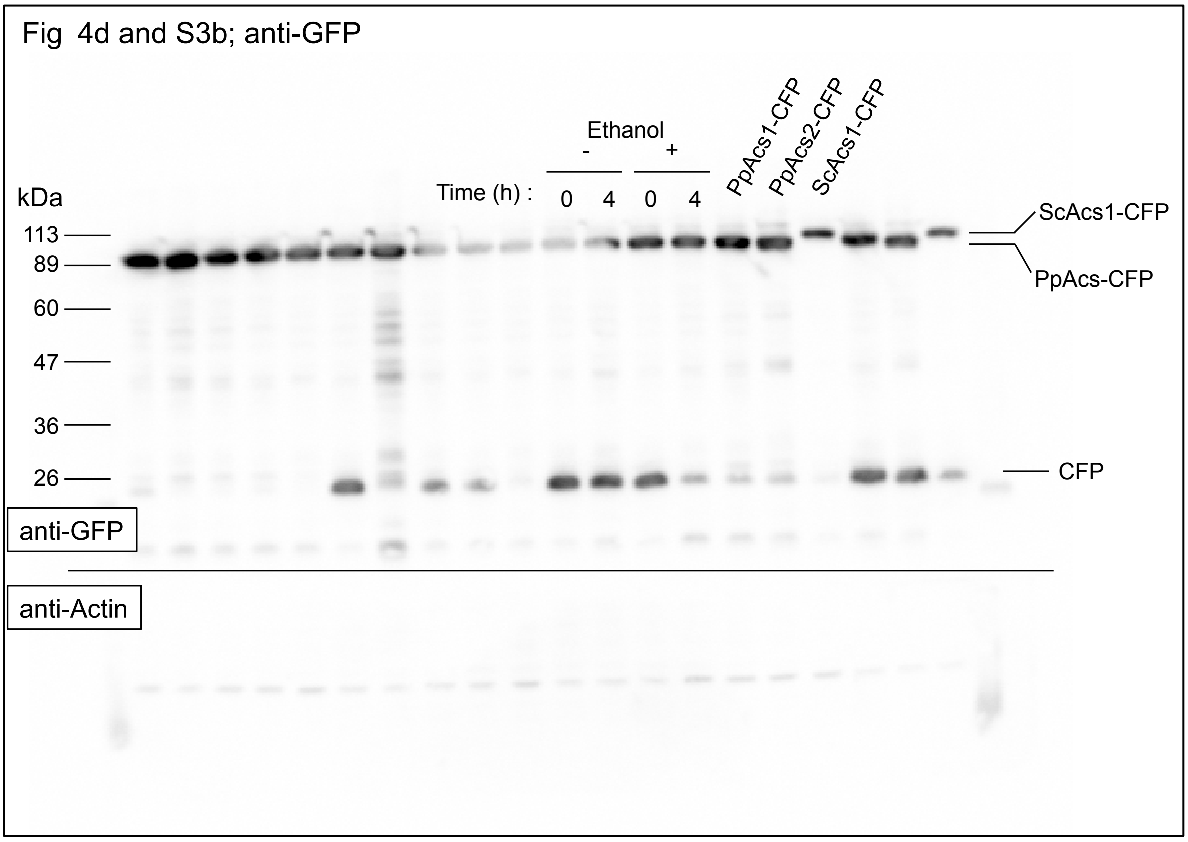

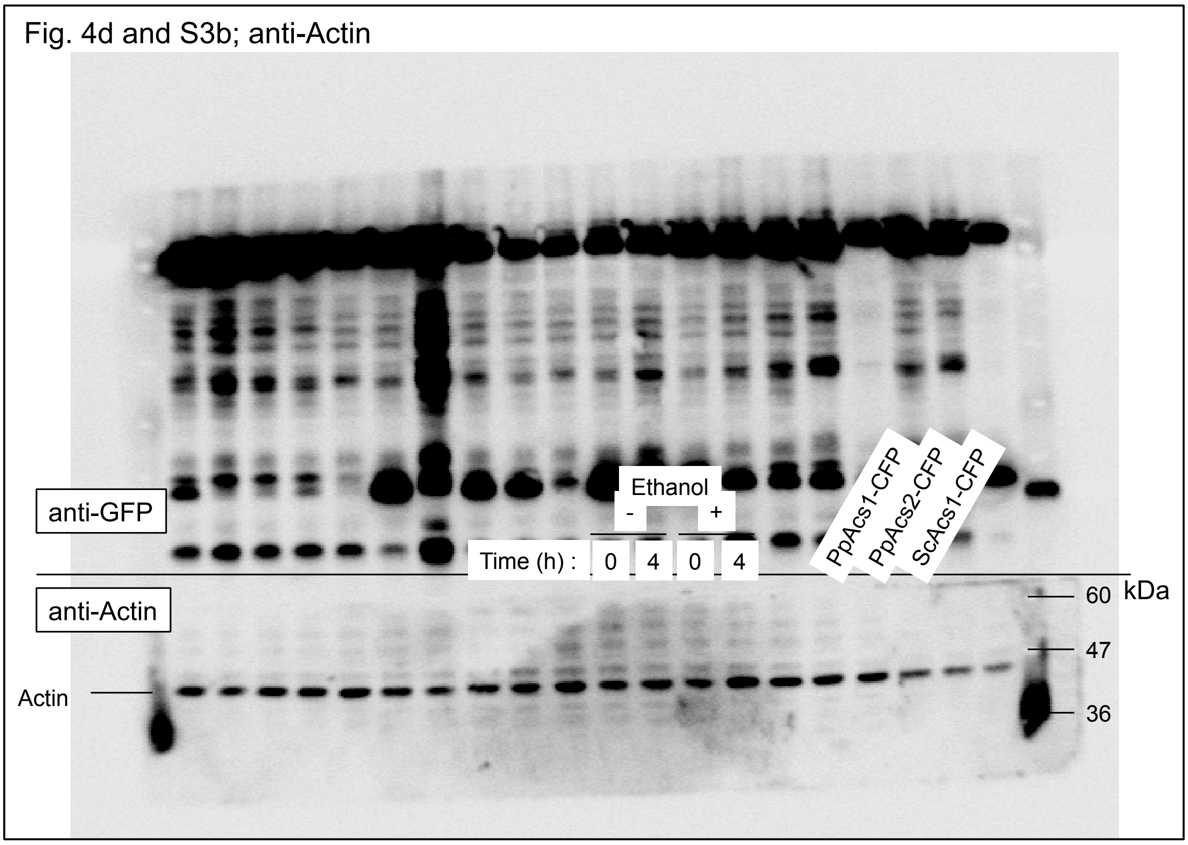

Supplementary Fig. S10

Fig. 4e; anti-GFP

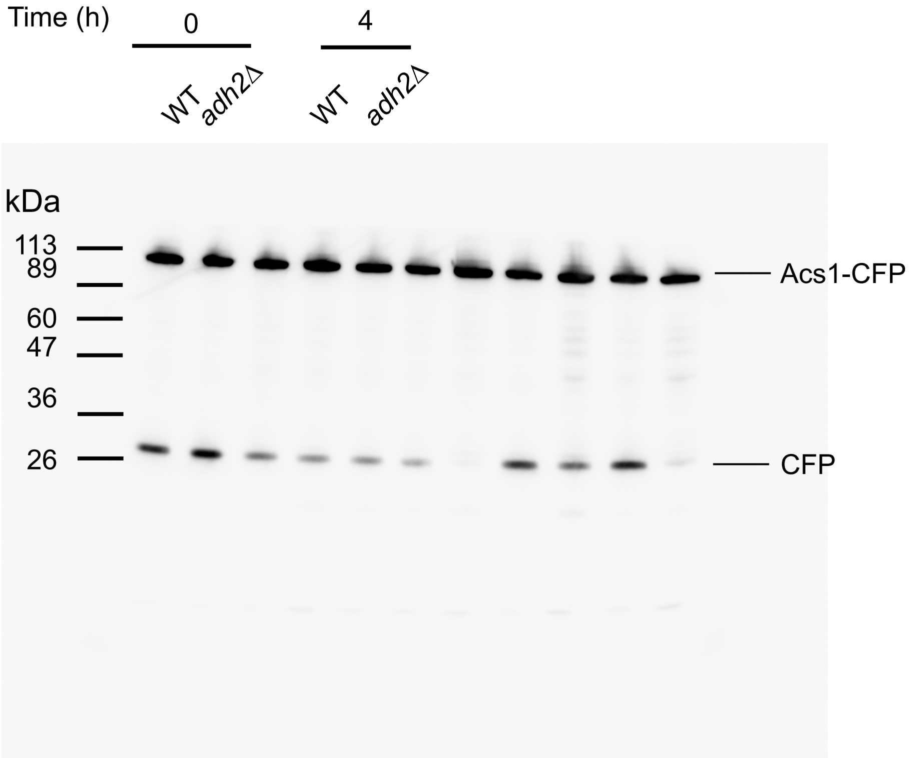

Fig. 4e; anti-Actin

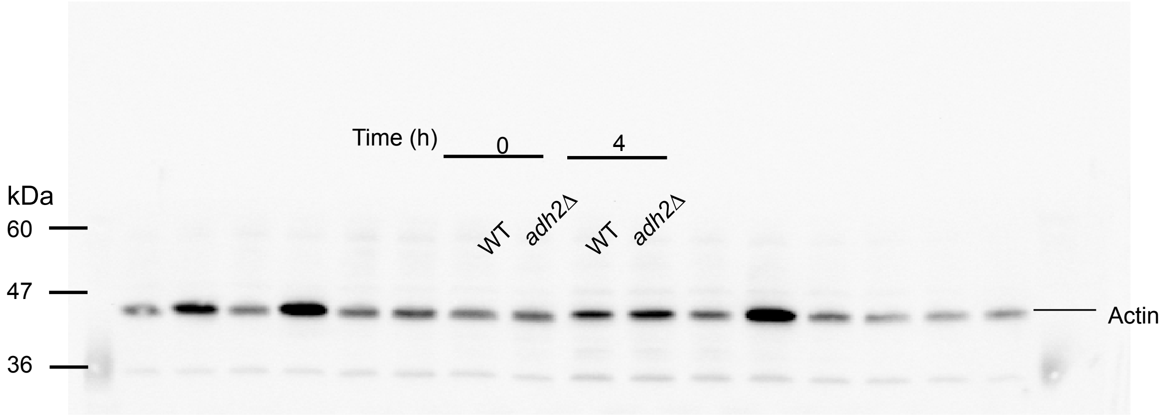

Supplementary Fig. S11

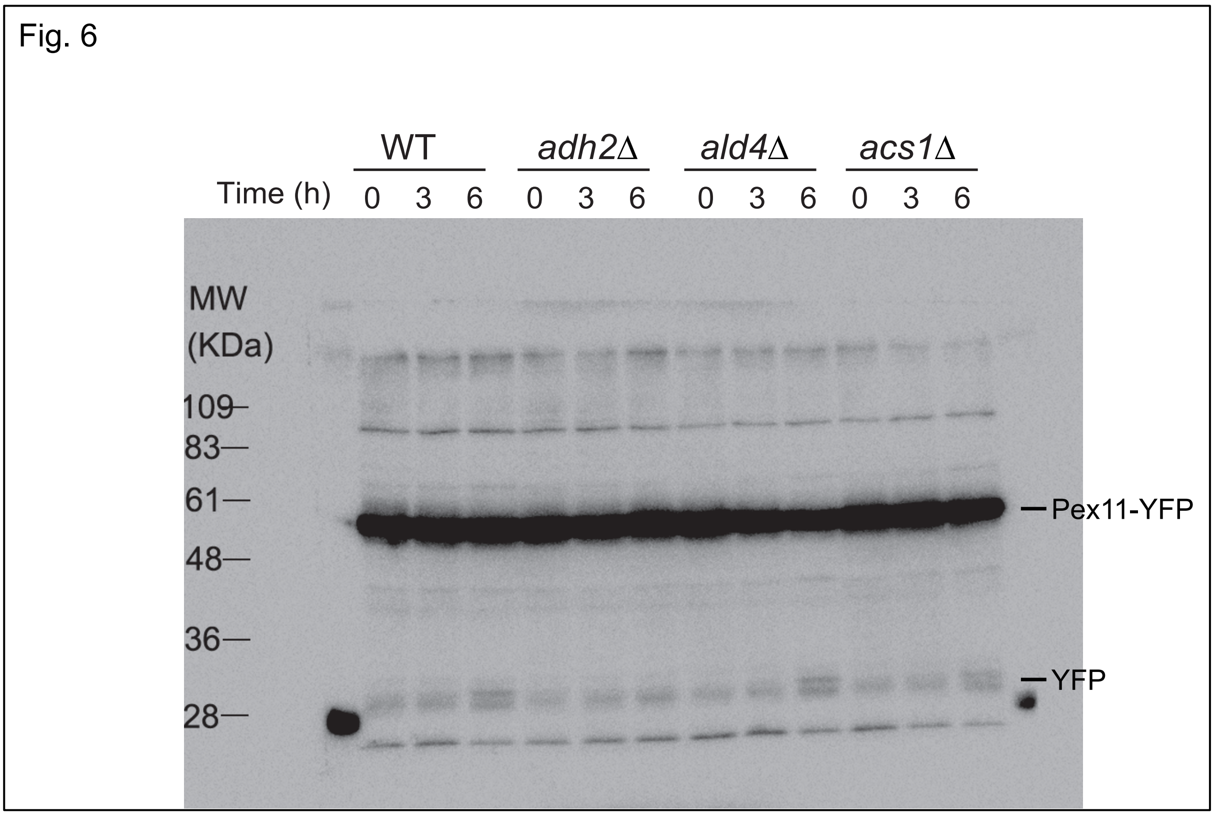

Supplementary Fig. S12

Supplementary Fig. S3d; anti-GFP

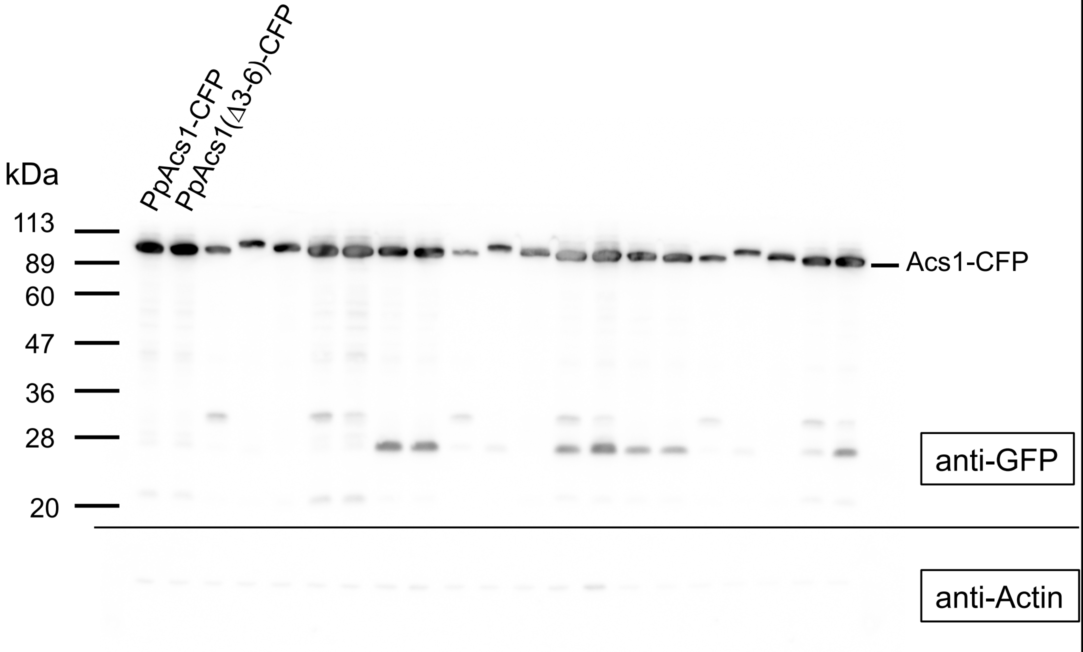

Supplementary Fig. S3d; anti-Actin

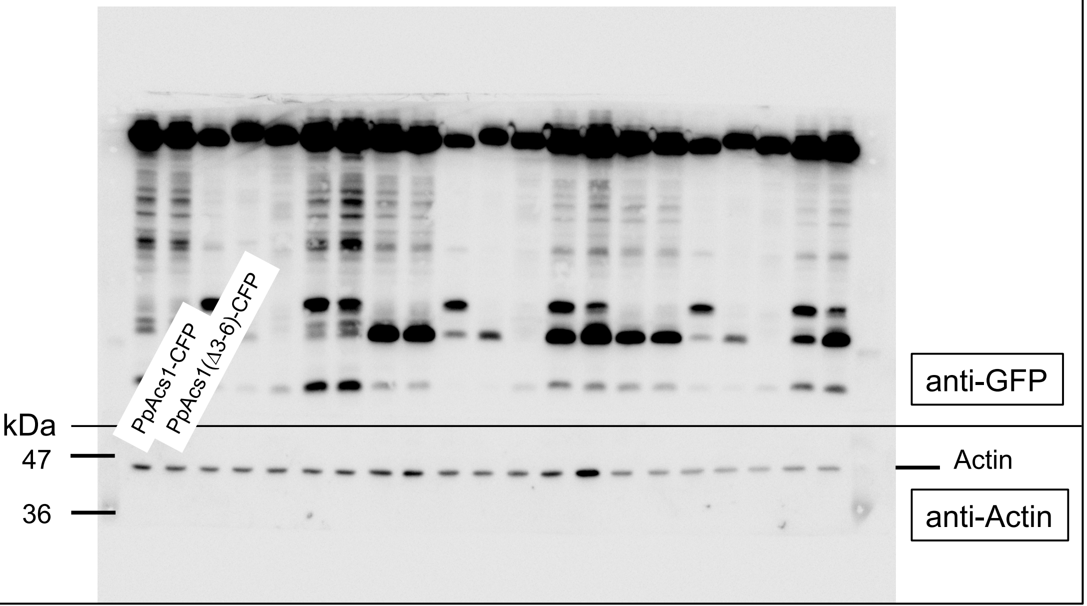

Supplementary Fig. S13

Supplementary Fig. S4a; anti-GFP & anti-AOX

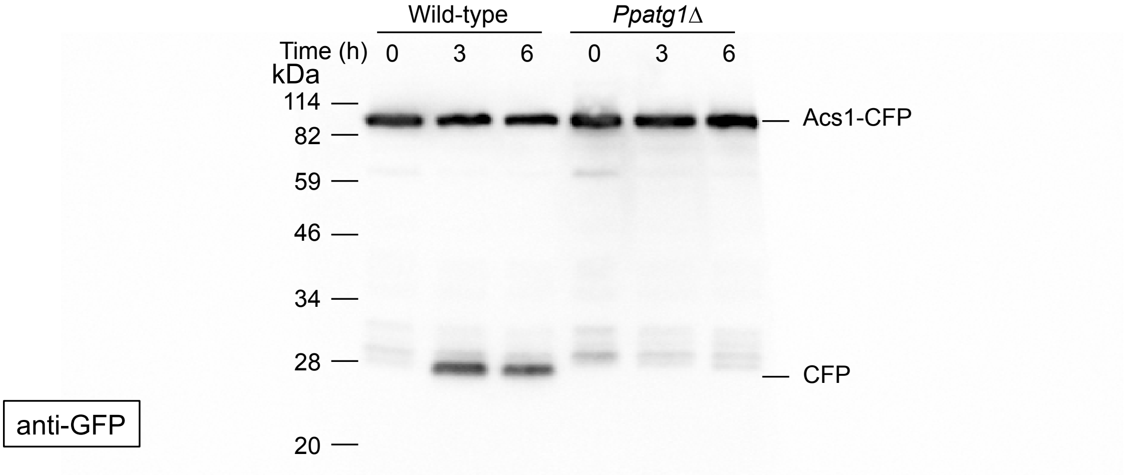

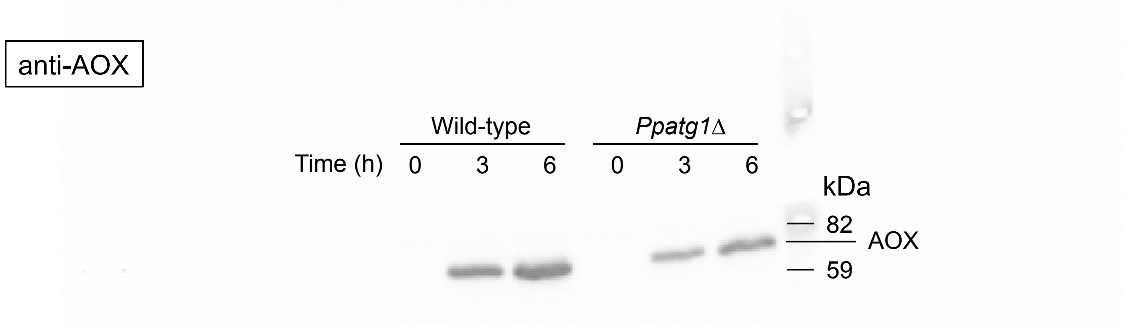

Supplementary Fig. S4a; anti-Actin

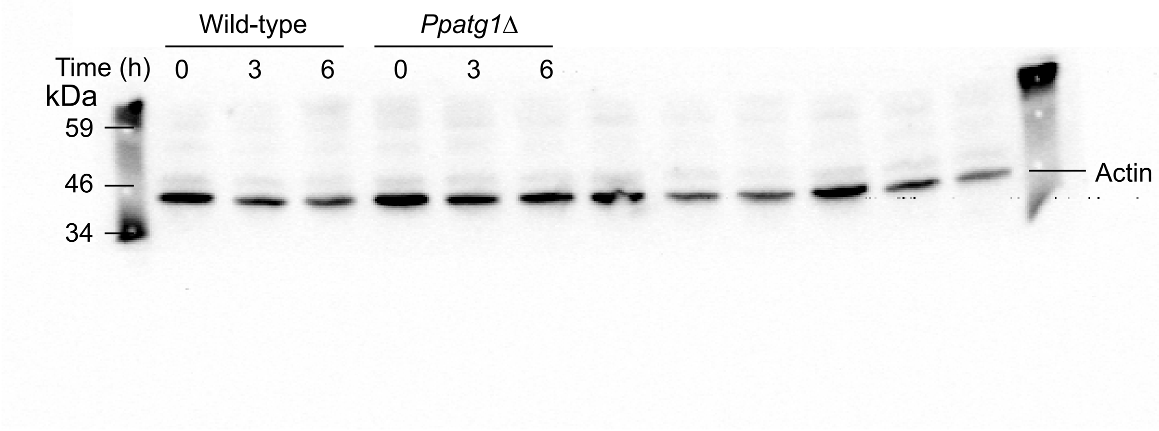

Supplement: Supplementary file 1 — Supplementary files [file 41598_2018_36732_MOESM1_ESM.pdf]
